# Evolutionary conservation of dopamine-mediated cellular plasticity in Arctic sponges (Porifera)

**DOI:** 10.3389/fmolb.2025.1671771

**Published:** 2025-11-17

**Authors:** Oksana I. Kravchuk, Alexander D. Finoshin, Yulia O. Nikishina, Victoria I. Melnikova, Ilya V. Kublanov, Dmitry A. Sutormin, Anastasiia N. Rusanova, Maxim T. Ri, Artem B. Isaev, Kirill V. Mikhailov, Rustam H. Ziganshin, Kim I. Adameyko, Anastasia A. Anashkina, Vasilina M. Ignatyuk, Nikolai G. Gornostaev, Elena E. Voronezhskaya, Agniya M. Sokolova, Victor S. Mikhailov, Yulia V. Lyupina

**Affiliations:** 1 N. K. Koltsov Institute of Developmental Biology, Russian Academy of Sciences, Moscow, Russia; 2 Winogradsky Institute of Microbiology, Federal Research Center of Biotechnology, Russian Academy of Sciences, Moscow, Russia; 3 Institute of Environmental Sciences, Hebrew University of Jerusalem, Rehovot, Israel; 4 Skolkovo Institute of Science and Technology (Skoltech), The Center for Molecular and Cellular Biology, Moscow, Russia; 5 A. N. Belozersky Institute of Physical and Chemical Biology, Lomonosov Moscow State University, Moscow, Russia; 6 A. A. Kharkevich Institute for Information Transmission Problems, Russian Academy of Sciences, Moscow, Russia; 7 Mass Spectrometry Group, Shemyakin and Ovchinnikov Institute of Bioorganic Chemistry of the Russian Academy of Sciences, Moscow, Russia; 8 Engelhardt Institute of Molecular Biology, Russian Academy of Sciences, Moscow, Russia

**Keywords:** Arctic sponges, dopamine, catecholamines, stress response, dopaminylation, cytoskeleton proteins, plasticity

## Abstract

Dopamine is an evolutionarily ancient signaling molecule implicated in stress responses across the tree of life. The role of dopamine is well-documented in the nervous system of animals, yet in the early-branching animal lineage of sponges its utility is poorly understood. Arctic marine sponges inhabiting the tidal zone of the White Sea, with fluctuating seasonal ice cover and solute concentrations, exhibit remarkable physiological plasticity, making them ideal models for studying conserved stress-response mechanisms. We investigated the dopamine signaling in two sponge species, *Sycon ciliatum* (class Calcarea) and *Halisarca dujardini* (class Demospongiae), using metagenomics, transcriptomics, high performance liquid chromatography, mass spectrometry, molecular docking, and immunofluorescence. *S. ciliatum* expresses an aromatic amino acid decarboxylase-like enzyme and efficiently converts L-DOPA to dopamine, whereas *H. dujardini* lacks this canonical biosynthetic enzyme, but accumulates dopamine, likely via its symbionts. During morphogenetic transitions in *H. dujardini*, genes involved in dopamine turnover, including tyrosinase, dopamine β-hydroxylase, and G protein–coupled receptors (GPCRs), showed dynamic expression. Molecular docking revealed that GPCR affinity for dopamine is modulated by cellular redox status. Notably, we report the first evidence of post-translational dopaminylation of cytoskeleton proteins in a non-bilaterian animal. Fluctuations in cellular dopamine levels and actin dopaminylation correlated with structural remodeling of the aquiferous system throughout the sponge life cycle. These findings demonstrate that dopamine regulates cellular plasticity through both transcriptional and post-translational mechanisms. The discovery of dopaminylation in sponges expands the evolutionary scope of catecholamine signaling and underscores the ancient role of dopamine in the regulatory interactions of animal cells.

## Introduction

Living organisms perceive and store information necessary for their survival using biomolecules sensitive to changes in the environment. The amino acids, including aromatic ones, containing a benzene ring as part of their side chain (tryptophan, phenylalanine and tyrosine), form the basis for present-day protein synthesis in living organisms. The aromatic acids, due to their structural features, stabilize protein molecules and quickly respond to external influences, allowing them to protect against UV radiation and increased concentrations of reactive oxygen species (ROS). They are considered to be essential even outside of their proteinogenic role (Han et al., 2019; Wang and Douglas, 1999), and their derivatives are often involved in the cell signaling and defense. Evolution of amino acid metabolism and the evolvement of catechol (a benzene ring with two hydroxyl (-OH) groups attached at adjacent (ortho-) positions) and catecholamines (derived from catechol by adding an amine group) are deeply tied to the development of the biochemical signaling systems responding to oxygenation (Fredrickson et al., 2022). Catechol has redox properties and can easily donate or accept electrons, making it useful for antioxidant activity and ROS detoxification (Schweigert et al., 2001). The amino acids glutamate and glycine, and monoamines (dopamine (DA), noradrenaline (NA), adrenaline (A), serotonin (5-HT), histamine (H)) are signaling molecules found in all domains of life (Moroz et al., 2021). Catechol groups in DA and flavonoids scavenge ROS via the redox cycle, protecting cells from oxidative damage (Kim et al., 2019). Additionally, in marine environments, catechol binds transition metals (e.g., Fe^2+^, Cu^2+^) thus preventing generation of free radicals by the Fenton reaction. Marine mussels exploit the metal-binding and cross-linking properties of catechol by secreting 3,4-Dihydroxyphenylalanine (DOPA)-rich adhesive proteins for attachment to substrates. DA exhibits strong adhesive properties and interacts with both organic and inorganic surfaces via covalent and non-covalent bonds (Ding et al., 2016; Lee et al., 2007). However, unpolymerized DA is cytotoxic and requires tight regulation of its levels and activity. Dopamine receptors are widespread in animals and belong to the large rhodopsin-like GPCR family. In humans five dopamine receptors are classified into D1-like receptors (D1 and D5) that stimulate adenylyl cyclase activity, and D2 -like receptors (D2, D3, and D4) that inhibit it. DA and is important for the functioning of the nervous system, but are also critical for vascular smooth muscle contractility and renal sodium transport in mammals (Fung et al., 2009; Mishra et al., 2019; Wang et al., 2020; Liu et al., 2023). Tyrosinase is present in all domains of life and catalyzes the conversion of tyrosine to dopaquinone (DAQ), initiating the polymerization of melanin (Aguilera et al., 2013). Melanin plays an important role in pigmentation and protection from UV radiation (Sarna and Swartz, 2006). The identification of monoamines in both animal and unicellular organisms underscored the deep evolutionary roots of this signaling system, revealing that their signaling functions are not exclusive to neurons, but rather an ancient feature of biological regulation. In the last decades the research has highlighted a regulatory process called monoaminylation, in which monoamines chemically modify proteins, mediated by transglutaminase-2 (Walther et al., 2011; Zheng et al., 2025). In mammals, DA has been shown to modify brain and cancer cell proteins (Lepack et al., 2020; Zhang et al., 2024). Meanwhile, the processes of DA signaling and alternative pathways of DA regulation in basal animals have not been studied sufficiently.

Sponges (Phylum Porifera) are aquatic filter feeders with a complex aquiferous system composed of multiple specialized cell types, some of which retain pluripotency (Sogabe et al., 2019; Lyupina et al., 2025). One of the most remarkable features of sponges is their ability to undergo cell reaggregation following body dissociation into individual cells and restoration of functional organization (Wilson, 1907). Sponge cells interact with symbiotic microbial communities, which influence the developmental processes through signaling molecules such as nitric oxide and DA. The DA derivatives, such as N-acyl dopamine glycosides and N-acyl dehydrotyrosine glycoside, that are structurally similar to the neurotransmitter and endocannabinoid N-arachidonoyl dopamine were isolated from the Icelandic seawater sponge *Myxilla incrustans* (cl. Demospongia) (Einarsdottir et al., 2016). Sponge cells express genes for G protein-coupled receptors (GPCRs) similar to other metazoans (Xiang et al., 2023). Studies have shown that bacterial monoamines can modulate larval behavior in *Amphimedon queenslandica* via rhodopsin-like GPCRs (Xiang et al., 2023). Although studies of the role of DA in sponges are limited, the symbiotic relationship and genomic complexity of sponges suggest the presence of DA-mediated regulatory mechanisms. Arctic marine sponges in the intertidal zone of the White Sea demonstrate significant physiological plasticity, enabling them to adapt to dynamic environmental conditions. The White Sea, a shallow semi-enclosed basin in the northern Russia, undergoes seasonal ice formation, which increases the concentration of dissolved substances (Cobelo-García et al., 2006). These characteristics make Arctic sponges a valuable model for studying the conserved mechanisms of cellular plasticity and stress response. The aim of this work was to identify DA-mediated mechanisms of plasticity of the cold-water sponges. We used High performance liquid chromatography (HPLC) to detect DA in the sponges *Sycon ciliatum* (Calcarea) and *Halisarca dujardini* (Demospongiae), demonstrating that despite the absence of canonical enzymes required for DA biosynthesis, sponges and their symbiotic bacteria may still produce this mediator through metabolic interactions with associated microbial partners. Analysis of transcriptomic data from the cold-water sponge *H. dujardini*, collected at different stages of the life cycle and during cell reaggregation following mechanical dissociation of the sponge body, was used to reveal differentially expressed genes involved in DA turnover. Mass spectrometry and immunofluorescence staining were used to show that DA signaling in sponge cells can be mediated via posttranslational modification of cytoskeletal proteins by dopaminylation. Analysis of DA signaling mechanisms in sponges reveals evolutionarily conserved features and reinforces the notion that catecholamines are integral to the physiological regulation of early-diverging animal lineages.

## Materials and methods

### Specimen collection

The sponge specimens were collected in the sublittoral zone of the White sea near the White Sea Biological Station of Moscow State University (66°34′N, 33°08′E) and Polar Circle tourist Center (66° 30′N, 33° 08 E): *H. dujardini* (cl. Demospongiae) (1–2 m depth) and *S. ciliatum* (cl. Calcarea) (2–5 m depth). Sampling of mature *S. ciliatum* was conducted in Spring (April) and *H. dujardini* in Winter (December, January, and February) from under the ice, Spring (March, April, and May), Autumn (September, November) (Supplementary Figure S1). All Arctic sponge *H. dujardini* specimens were collected from the natural population in the White Sea, at consistent depth and location across seasons. Due to our commitment to minimal environmental impact, sponges were collected individually with strict adherence to conservation guidelines. Winter collections required ice-hole diving at a distance from the main site to avoid disturbance. To preserve the natural microenvironment, sponges were collected together with their substrate (macroalgae) and maintained as described earlier (Adameyko et al., 2021).

Sponge dissociation and reaggregation experiment was made as described earlier (Finoshin et al., 2020). Sponge tissue was cut into small fragments in a Petri dish containing filtered seawater (FSW; 10% w/v). The resulting cell suspension was gently triturated five to ten times using a micropipette fitted with a 1000-μL tip and then passed through a 40-μm nylon mesh. The filtrate was centrifuged at 300 *g* for 5 min at 10 °C–12 °C. Cell concentration and viability were assessed using a hemocytometer after mixing 10 μL of cell suspension with 10 μL of 0.4% trypan blue; viability consistently exceeded 96%–98%. Dissociated cells were used for downstream analyses within 20 min of preparation.

For the L-DOPA treatment experiment, sponges, collected in Autumn (November) (on macroalgae, to save their structural and functional integrity for filtration) were incubated for 2 h at +8 °C with 10 μM L-DOPA solution prepared in filtered seawater (FSW), while the control sponge was incubated in FSW alone. After 2 h of incubation the sponges were placed in FSW for 20 min and prepared for HPLC analysis.

### High performance liquid chromatography with electrochemical detection (HPLC-ED)

Tissue samples were quickly dissected on ice, homogenized with an ultrasonic homogenizer (BandelinSonopuls, Burladingen, Germany) at 4 °C in 0.1 M HClO_4_ (10% w/v), containing 3,4-dihydroxybenzylamine (DHBA) as an internal standard and centrifuged at 10,000 g for 20 min at 4 °C. The supernatants were collected and stored at −80 °C prior to measurements of catecholamine content. Just before the analysis, catecholamines were extracted by the alumina-based purification method (Anton and Sayre, 1962). Briefly, approximately 15 mg of activated AI_2_0_3_ were added to 1 mL of homogenate followed by 250 μ^1^ of 1M TRIS buffer (pH 8.8). Samples are extracted by shaking for 10 min at 8 °C followed by a brief centrifugation (500 g, 3 min) to pellet the A1_2_0_3_. The aqueous layer was aspirated and the A1_2_0_3_ was rinsed with 1 mL of 0.01 M Tris buffer (pH 8.8), gently vortexed, centrifuged and the buffer thoroughly aspirated. Catecholamines were then desorbed from the A1_2_0_3_ by the addition of 100 μ^1^ of 0.2 N HC10_4_ and shaking for 10 min followed by brief centrifugation. An Agilent 1,260 Infinity II HPLC system (Agilent Technologies Inc., Waldbronn, Germany) was used for the catecholamine measurement. Electrochemical detector Decade Elit (Antec, the Netherlands) was equipped with a glass carbon flow cell and salt-bridge Ag/AgCl reference electrode with the potential set at +0.85 V. Analytes were separated using a reverse-phase InfinityLab Poroshell 120 EC-C18 100 mm × 4.6 mm column with a 2.7 μm particle size (Agilent Technologies Inc., Germany). The column was thermostatted at 29 °C. The mobile phase consisted of 0.1 M citrate–phosphate buffer, 0.25 mM 1-octanesulfonic acid sodium salt, 0.1 M EDTA, and 7% acetonitrile (pH = 2.56) (all reagents purchased from Sigma-Aldrich, St. Louis, MO, USA). The mobile phase flow rate was 1 mL/min. Peaks of catecholamines were identified by the retention time relative to the standard solutions, and the contents of substances were estimated as a ratio of the peak area of the internal standard solution to that of the specimen.

### Phylogenetic analysis

The datasets for phylogenetic analysis of DOPA decarboxylases, aromatic amino acid hydroxylases, tyrosinases, DBH-like monooxygenases, and the rhodopsin-like GPCRs were assembled using the orthology inference of OrthoFinder (Emms and Kelly, 2019) with a selection of eukaryotic, primarily metazoan genomes. For the DOPA decarboxylase dataset three orthogroups containing animal group II pyridoxal-dependent decarboxylases were combined and supplemented with a selection of prokaryotic sequences from the related COG (Galperin et al., 2025): COG0076 - Glutamate or tyrosine decarboxylase or a related PLP-dependent protein. The DOPA decarboxylase dataset and orthogroups of aromatic amino acid hydroxylases, tyrosinases, and DBH-like monooxygenases were additionally expanded with sponge homologs acquired by BLAST (Altschul, 1997) searches with the NCBI’s *nr* database (Sayers et al., 2025). The dataset for rhodopsin-like GPCRs was constructed using Pfam domain searches in a selection of animal genomes, and by combining several orthogroups containing members with the rhodopsin family receptor domain (PF00001). The selected species have well-annotated genomes and provide a suitable reference for inferring the relationship of *H. dujardini* GPCR family members to known subfamilies of neurotransmitter receptors. The sequences were aligned using MAFFT (Katoh and Standley, 2013) with the L-INS-i algorithm, and trimmed using trimAl (Capella-Gutierrez et al., 2009) with a gap threshold of 0.3 to remove alignment regions consisting mainly of gaps. The phylogenetic reconstruction was carried out using the Maximum Likelihood method of IQ-TREE (Minh et al., 2020) with automatic model selection (Kalyaanamoorthy et al., 2017) and node support evaluated using ultrafast bootstrap (Hoang et al., 2018) with 1,000 replicates. The reconstructed phylogeny was visualized using the MEGA software (Kumar et al., 2018) and the iTOL online tool (Letunic and Bork, 2024). The domain architectures were identified and drawn using the SMART resource (Letunic et al., 2021).

### *H. dujardini* metagenomic DNA extraction and sequencing

Two samples of *H. dujardini* were used for total metagenomic DNA extraction for short-read sequencing (the Hal sample). DNA was extracted from 0.5 g of fresh *H. dujardini* body tissue using DNeasy PowerLyzer Microbial Kit (Qiagen, Hilden, Germany) according to the manufacturer’s instructions, including a bead-beating stage using a FastPrep-24™ 5G grinder (MP Bio, Santa Ana, CA, USA). For bacterial fraction enrichment, a protocol for nuclei isolation was adapted (for the Hal-pro sample) (Gaublomme et al., 2019). Briefly, 0.5 g of fresh *H. dujardini* tissue was transferred to sterile prechilled 35 mm Petri dish on ice. The tissue fragment was minced in 1 mL of ice-cold Lysis Buffer (10 mM HEPES pH 7.2, 5 mM MgCl2, 10 mM NaCl, 0.005% NP40) using two disposable scalpels. Tissue was triturated by 10x pipetting with 1 mL tip and transferred to MACS C-tube. Additionally, the Petri dish was rinsed with 1 mL Lysis Buffer, and it was transferred to C-tube. The tissue was homogenized using gentleMACS Program “h_mito_01” in the cold room. The homogenate was incubated for 10 min on ice and homogenized again using the same program. An equal volume (2 mL) Isotonic Adjustment Buffer (10 mM HEPES pH 7.2, 5 mM MgCl2, 10 mM NaCl, 500 mM Sucrose) was added to MACS C-tube and mixed with homogenate. The homogenate was filtered through a pre-wetted (with 200 μL Isotonic Wash Buffer - 10 mM HEPES pH 7.2, 5 mM MgCl2, 10 mM NaCl, 250 mM Sucrose, DPBS +1% BSA) 70 μm filter into a 15 mL tube. 2 mL Isotonic Wash Buffer was added to MACS C-tube, rinsed around the tube and then rinsed through the filter. The filtered homogenate was centrifuged at 1000 *g* for 5 min at 4 °C. The supernatant was collected (the bacterial fraction) and the pellet (nuclei fraction) was discarded. Bacteria were pelleted by centrifugating the bacterial fraction at 5000xg for 5 min at 4 °C. DNA extraction was performed from the bacterial pellet using DNeasy PowerLyzer Microbial Kit (Qiagen, Hilden, Germany) according to the manufacturer’s instructions, including a bead-beating stage using a FastPrep-24™ 5G grinder (MP Bio, Santa Ana, CA, USA).

A shotgun WGS library preparation was performed for Hal and Hal-pro samples at Laboratory “Genomed” Ltd. (Moscow, Russia) using the MGIEasy Fast PCR-FREE FS Library Prep Set (MGI, China) according to the manufacturer’s protocol. Sequencing was performed using the DNBSEQ-G400 system (MGI, China) in a 150 + 150 nt paired-end mode.

For nanopore sequencing (the Nanopore sample), genomic high-molecular-weight (HMW) DNA was extracted from 1 g of *H. dujardini* tissue, using the phenol-chloroform method following standard protocols (Rusanova et al., 2022), with additional purification performed using the CTAB method to ensure high-quality DNA (Wörheide et al., 2024). The DNA was further size selected using the Circulomics Short Read Eliminator (SRE) Kit, following the manufacturer’s protocol, to enrich for long DNA fragments. Sequence libraries were prepared using the Ligation Sequencing Kit SQK-LSK109 (Oxford Nanopore), following the manufacturer’s protocol. Sequencing was conducted for 48 h using a MinION Flow Cell (FLO-MIN106D, R9.4.1 version; Oxford Nanopore). The MinKNOW software package (version 19.12.5) was used for basecalling and demultiplexing the reads. Sequencing was performed three times, and the resulting data were combined for analysis. Sequence data was deposited to NCBI Sequencing Read Archive (SRA) under a BioProject ID PRJNA1347440.

### Metagenome assembly, binning, and refinement of bins

For Illumina-generated short-read shotgun datasets Hal and Hal-pro, the quality of raw sequencing reads was initially assessed using FastQC (Andrews et al., 2012). Adapter sequences and low-quality reads were trimmed using Trimmomatic v0.39 with parameters PE -phred33 ILLUMINACLIP:2:30:10 (Bolger et al., 2014). Survived paired-end and forward unpaired reads were used for *de novo* cross-assembly with SPAdes v3.15.4 in the--meta mode and the k-mer sizes of 33, 55, and 99 (Nurk et al., 2017).

Short reads (combined Hal and Hal-pro) were aligned to a metagenome assembly using bwa-mem2 (Vasimuddin et al., 2019). Metagenome assembly was binned using MaxBin 2.0 (Wu et al., 2016), CONCOCT (Alneberg et al., 2014), MetaBAT2 (Kang et al., 2019), and binny (Hickl et al., 2022). Quality of obtained metagenomic bins was evaluated using QUAST (Gurevich et al., 2013) and CheckM2 (Parks et al., 2015), and bins with completeness >90% and contamination <5% were retained for further analysis. Selected bins generated by different binners were classified using GTDB-Tk v2.4.1 classify_wf function with GTDB database release 226 (Chaumeil et al., 2022) for dereplication. Bins classified identically by GTDB were collected and refined with CORITES (Rusanova et al., 2025) using long-read ONT sequencing data (the Nanopore sample), which were preprocessed using Porechop (--barcode_threshold 90) (Wick et al., 2017). For the Eutrophobiaceae bins, CORITES parameters mq_thr = 30 and br_thr = 9 were used. Bins refined by CORITES were additionally scaffolded using Longstitch (Coombe et al., 2021). Additionally, for the Casp-alpha2 bin, we used a previously obtained *H. dujardini* genome sequences (Zubarev et al., 2025 (https://zenodo.org/records/14981466, SKOL_HDuj_1.0.fasta). Resultant metagenome-assembled genomes (MAGs) were deposited to NCBI GenBank under a BioProject PRJNA1347440.

### Functional annotation of metagenomic bins

Reconstructed MAGs and bins, obtained with binny, were annotated using Prokka (Seemann, 2014). Predicted proteins were further annotated using BlastKOALA v. 3.0 (Kanehisa et al., 2016) and biosynthetic pathways were inspected using KEGG mapper (Kanehisa and Sato, 2020). Phylogenetic trees were constructed using GTDB-Tk v2.4.1 de_novo_wf with GTDB database release 226 (contains 715,230 bacterial genomes) (https://doi.org/10.1093/nar/gkab776) (https://doi.org/10.1093/bioinformatics/btac672) and visualized with iTol v6 (Letunic and Bork, 2024).

### Docking analysis

The protein structures *H. dujardini* G-protein coupled receptors were modeled in MOE 2019.0102 (Molecular Operating Environment, 2019, Canada) using homology modeling based on the AlphaFold structures AF-A0A1X7U348-F1, AF-A0A1X7U4C3-F1, AF-A0A1X7UKS4-F1 and AF-A0A7M5V649-F1-v4 as structures of closest homologs from *A. queenslandica* and *Clytia hemisphaerica*. Docking analysis was performed using Amber10 force field in MOE program with ligands DA, NA and 5-HT both in blind and site finder modes. A human dopamine receptor D1 with a known crystal structure (PDB id 7LJD) was used as a reference protein. Binding pocket was selected by the best ligand affinity value to potential binding sites defined by MOE program. Docking was performed by Triangle Matcher placement method with Rigid Receptor refinement, London dG and GBVI/WSA dG scores, with 30 start docking poses and five best final docking variants.

### Identification of monodansyl cadaverine binding

Clarified cell lysates containing native proteins with protease inhibitors were prepared as previously described (Adameyko et al., 2021). Equal volumes of lysate were distributed into separate tubes. Different samples were treated with 1 mM calcium chloride to activate transglutaminase; 100 µM cystamine dihydrochloride (Sigma/Aldrich) to inhibit transglutaminase activity; or buffer alone to equalize protein concentrations across samples. Monodansyl cadaverine (MDC, Sigma Aldrich) at a final concentration of 10 µM were added to all tubes except the controls 30–40 min after the other reagents were introduced, to ensure the inhibitors had sufficient time to take effect. Lysates wereincubated for 2 h at room temperaturAnd then lysates were loaded onto 10% polyacrylamide gels, and SDS-PAGE electrophoresis was performed for 30 min at 100 V followed by 1 h at 160 V in Tris/glycine/SDS running buffer.

After electrophoresis, the gels were transferred to a Bio-Rad ChemiDoc gel documentation system using appropriate excitation and emission settings, the SybrGreen channel was used. After imaging, the gel was stained with Coomassie Brilliant Blue R-250 for 15 min, followed by destaining in 7% acetic acid for 24 h.

### SDS-PAGE

Samples containing proteins were diluted in 2хSLB buffer and maintained for 4 min in a water bath at 95 °C. Polyacrylamide gels (10%) were loaded with samples (60 μg of protein/well), and SDS-PAGE electrophoresis was carried out for 30 min at 100V then 1 h at 160V in Tris/glycine/SDS running buffer as described earlier (Finoshin et al., 2020).

### Chromatography-mass spectrometric analysis

Lysates were subjected to electrophoresis followed by Coomassie staining. Protein bands corresponding to 41–42 kDa were excised using a sterile scalpel and transferred to sterile microcentrifuge tubes for storage at −80 °C until mass spectrometric analysis. Prior to LC–MS/MS, samples were reconstituted and loaded in loading buffer (2% acetonitrile, 98% H_2_O, 0.1% trifluoroacetic acid) at a flow rate of 4 μL/min onto a custom-made trap column (50 × 0.1 mm) packed with ReproSil-Pur 200 C18-AQ, 5 µm particles (Dr. Maisch), maintained at room temperature. Samples were subsequently eluted onto a home-packed fused-silica analytical column (300 × 0.1 mm) filled with ReproSil-Pur C18-AQ, 1.9 µm particles (Dr. Maisch), coupled to an emitter tip fabricated using a P-2000 laser puller (Sutter Instrument, USA). Reverse-phase chromatography was performed on an Ultimate 3,000 NanoLC system (Thermo Fisher Scientific) coupled online to an Orbitrap Tribrid Lumos mass spectrometer (Thermo Fisher Scientific) via a nanoelectrospray ion source (Lyupina et al., 2025). The mass spectrometry proteomics data have been deposited to the ProteomeXchange Consortium via the PRIDE (Perez-Riverol et al., 2025) partner repository.

### Analysis of mass spectrometric data

Mass spectrometric data were analyzed using PEAKS Studio 11.0 software (Bioinformatics Solutions Inc.). Protein identification was performed by matching tandem mass spectra to *H. dujardini* protein sequence database (NCBI, PRJNA594150) previously obtained by our group and *S. ciliatum* protein sequence database (Fortunato et al., 2015) (https://doi.org/10.5061/dryad.tn0f3). The following parameters were used: fixed modification–carbamidomethylation of cysteine; variable modifications–deamidation of asparagine/glutamine, oxidation of methionine, acetylation of protein N-term, acetylation of lysine, methylation, ubiquitination, serotoninylation of glutamine; conjugation of noradrenaline. The following modifications involving DA have been studied: dopaminylation of glutamine, dopamine quinone modification of lysine, 3,4-dihydroxyphenylacetaldehyde modification of lysine, dopaminochrome modification of lysine, dopamine quinone modification of cysteine, dopaminylation of cysteine (Supplementary Table S1). The false discovery rate (FDR) threshold for peptide identification was set at 0.01, determined using a decoy database of reversed amino acid sequences. Protease specificity was set to cleavage at the C-terminus of arginine and lysine residues, allowing for up to two missed cleavage sites. The precursor mass tolerance was set to 10 mDa, and the fragment mass tolerance was 0.05 Da.

### Western blotting

The *H. dujardini* body tissue probes running in 10% SDS-gel were transferred from the gel onto a 0.45-μm nitrocellulose membrane by wet electroblotting at 100 V for 60 min in a transfer buffer composed of Tris, glycine, and 30% methanol. Transfer efficiency was verified by staining the membranes with Ponceau S solution. Following this, the membranes were blocked overnight at 4 °C with 5% non-fat milk in Tris-buffered saline containing Tween-20 (TBST). Then they were incubated overnight at 4 °C with primary antibodies diluted in blocking buffer: polyclonal rabbit anti-DA antibodies (Abcam ab6427, Enzo Life Sciences BML-DA1140) at dilution 1:500 or monoclonal mouse anti-β-actin (ACTBD 11B7, Santa Cruz Biotechnology) at 1:1,000. After washing with TBST three times for 10 min, membranes were incubated for 1.5 h at room temperature with the second antibodies: Alexa Fluor 546 goat anti-rabbit IgG or Alexa Fluor 633 donkey anti-mouse IgG (Invitrogen/Thermo Fisher Scientific, Waltham, MA, USA, (1:800)). After washing with TBST three times for 10 min the immunoreactive bands were visualized using enhanced fluorescence detection and imaged on a GelView 6000Pro II Imaging System (Biologist Biotechnology Co., Ltd, Guangzhou, China) equipped with a specific filter. The Western blot analysis was used to verify DA antibody binding for immunofluorescence study.

### Immunofluorescent cell staining

The immunofluorescent staining with the DA and actin antibodies was performed for *H. dujardini* samples to assess DA and actin distribution within the cells. The monoclonal mouse ACTBD 11B7 (1:1,000)) antibodies or polyclonal rabbit A2066 (Sigma, Israel, (1:500)) against β-Actin or phalloidin Alexa 546 (Invitrogen/Thermo Fisher Scientific, Waltham, MA, USA, (1:800)) were used for the assessment of the subcellular localization of protein with polyclonal rabbit anti-dopamine antibodies (Abcam ab6427, (1:500) or mouse mAb anti-Glu-Lys link antibody (ab424, Abcam, Cambridge, United Kingdom, (1:150)). To reveal the actin distribution in sponge body tissue, individual specimens were fixed with 3.5% paraformaldehyde solution (in FSW) for 2–6 h at RT, washed in PBS, and embedded in cryoprotectant. Freezing was carried out at −40 °C in hexane. Sections from three or four independent sponge samples, each with a thickness of 12 μm, were cut using a Leica CM1950 cryostat and mounted onto the same adhesive glass slides (*n* = 8). Sections were dried, rehydrated in PBS and stained overnight with antibodies against actin and dopamine or anti-Glu-Lys link antibody prepared in PBS with 0.5% Triton X-100% and 5% fetal bovine serum (FBS). After washing with PBS three times for 10 min, samples were incubated for 2 h at 20 °C with the second antibodies: Alexa Fluor 546 goat anti-rabbit IgG or Alexa Fluor 633 donkey anti-mouse IgG (Invitrogen/Thermo Fisher Scientific, Waltham, MA, USA, (1:800)) prepared in PBS with addition of 0.3% Triton X-100% and 5% FBS, phalloidin was added in this period. The control slides were stained overnight only with PBS with 0.5% Triton X-100% and 5% FBS and then with secondary antibodies. The Hoechst-33342 (Invitrogen/Thermo Fisher Scientific, Waltham, MA, USA) staining for the nucleus was done for 5 min after incubation with the second antibodies. Then, the slides were washed in PBS and placed in Mowiol (Calbiochem/Merck KGaA, Darmstadt, Germany) for confocal images using the Carl Zeiss LSM 880 laser scanning microscope (Carl Zeiss AG, Oberkochen, Germany) equipped with a 63 × /1.4 oil immersion Plan-Apochromat objective. Image acquisition was performed with Airyscan technology in Z-stack mode. Fluorescence signals were recorded with excitation at 543 nm for phalloidin, 633 nm for DA, and 405 nm for nuclear staining with Hoechst-33342. Emission detection ranges were adjusted to minimize spectral overlap. The pinhole was set to 1 Airy unit, corresponding to an optical section thickness of ∼0.9 μm. Z-stacks were collected at step intervals of 0.5 μm. Scanning was performed at a resolution of 1,024 × 1,024 pixels with line averaging set to eight to enhance the signal-to-noise ratio. Images were acquired and processed using Zeiss ZEN Black software, with Airyscan processing applied. Only linear adjustments of brightness and contrast were applied uniformly across the dataset.

### RNA-Seq differential expression analysis

We used our previously published bulk RNA-Seq dataset (NCBI BioProject accession PRJNA594150), comprising *H. dujardini* samples collected at four time points over an annual cycle (Winter, Spring, Summer, and Autumn) and at three reaggregation stages (intact sponge body, cell suspension 30 min after dissociation, and cell aggregates 24 h after dissociation), and mapped it to our recently completed chromosome-scale genome of *H. dujardini* [https://zenodo.org/records/15992695; publication pending]. Quality control, read mapping, and differential expression analysis followed our earlier protocols (Lyupina et al., 2025). Briefly, fastp v.0.24 (Chen et al., 2018), STAR v.2.7.11 (Dobin et al., 2013), featureCounts v.2.14.2 (Liao et al., 2014), edgeR v.3.42.4 (Robinson et al., 2010), and ComplexHeatmap v.2.7.7 (Gu et al., 2016) were used. On average, libraries comprised 28.7 million of 50bp single-end reads after filtering, with 98.5% retained, and duplication rate averaged 62% (Supplementary Table S2). Each reaggregation stage has three replicates, except for Autumn samples and the Summer intact body, which retained two replicates after quality control. Alignment rates were high, with 94% total mapping and 73% uniquely mapped reads; GC content was stable at 51% (Supplementary Table S2), slightly higher than the genomic baseline of 43.98%, reflecting the presence of symbiotic transcripts. Because each season was sequenced in a separate batch, we modeled season as a biological covariate rather than removing it through full batch correction, which could obscure genuine biological variation. Batch-corrected counts generated with Combat-seq v.6 (Zhang et al., 2020 and Combat-ref v.1 (Zhang, 2024), were used only for PCA and sensitivity checks, confirming that stage-related changes were consistent across seasons (Supplementary Figure S2). As cross-season comparisons are qualitative, differential expression was assessed with a GLM quasi-likelihood model restricted to within-season contrasts (“cell suspension–intact body” and “cell aggregates–intact body”). P-values from all contrasts were pooled and adjusted with the Benjamini–Hochberg procedure, and significance thresholds were set at FDR 0.001 and absolute fold change 1.5 to balance statistical stringency with interpretability given modest replicate numbers (Supplementary Figure S3; Supplementary Table S3).

### Single cell RNA sequencing

The scRNA-seq data were obtained in an earlier study (Lyupina et al., 2025) and are available from the Zenodo: doi.org/10.5281/zenodo.14981466. The samples correspond to two individuals of the cold-water sea sponge *H. dujardini* that were collected at the end of August. Seurat 5.0.1 used for read count normalization, variable feature selection, scaling, dimension reduction and to cluster the cells. Approximation and Projection (UMAP) method was then used to visualize the clustering distances. Markers were identified using the FindAllMarkers function and subsequently used for cluster annotation (Lyupina et al., 2025).

### Statistical analysis

Analyses of HPLC data and the optical densities were performed using GraphPad Prism 8.1.1. For comparisons between two groups, an unpaired t-test was used when data passed the Shapiro–Wilk normality test; otherwise, the Mann–Whitney U test was applied. For multiple group comparisons, the Kruskal–Wallis one-way analysis of variance on ranks was used, followed by Dunn’s *post hoc* test. The Kruskal–Wallis one-way analysis of variance on ranks for multi-group comparisons does not assume equal variances or normality and is well-suited for unbalanced designs. Post-hoc pairwise comparisons were conducted using Dunn’s test, which is explicitly designed for use after Kruskal–Wallis when group sizes are unequal and controls the family-wise error rate appropriately. Pearson’s chi-squared test with Yates’ continuity correction by R version 4.4.2 (R Core Team (2024). R: A Language and Environment for Statistical Computing. R Foundation for Statistical Computing, Vienna, Austria, https://www.R-project.org/) were used for the estimation of the proportion of actin peptides in LC-MS data.

### Code availability

All code used to generate the single-cell atlases is deposited at Zenodo and GitHub: doi.org/10.5281/zenodo.14981466. https://github.com/kim-fehl/Halisarca-dujardinii-transcriptomics.

## Results

### High performance liquid chromatography (HPLC)

To assess the physiological significance of DA in marine sponges, we first quantified its endogenous levels using HPLC and reconstructed DA biosynthetic pathway in sponge using bioinformatic tools. To investigate whether DA and NA concentrations vary seasonally in the cold-water demosponge *H. dujardini* (cl. Demospongia)*,* specimens were collected across different seasons. The summer specimens of *H. dujardini* were not used for the HPLC experiments to avoid extra signals from cyanobacteria and microalgae.

**FIGURE 1:**
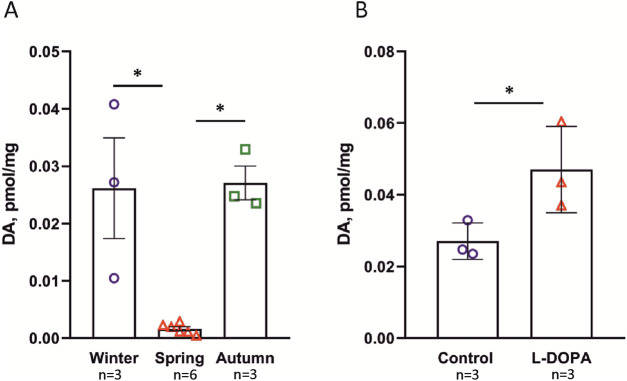
DA content in the cold-water sea sponge *H. dujardini* determined by HPLC. **(A)** Seasonal variation in DA levels (pM/mg tissue). Spring samples (*n* = 6) exhibited significantly lower DA concentrations compared to autumn (*n* = 3; *p* = 0.0221) and winter (*n* = 3; *p* = 0.0234), as determined by Kruskal–Wallis one-way ANOVA on ranks with Dunn’s *post hoc* test. The larger sample size for spring confirms the reproducibility of low spring DA levels and sharpens the contrast with the higher concentrations observed in autumn and winter. **(B)** The effect of L-DOPA on DA level. DA content increased significantly in the presence of 10 μM L-DOPA (*p = 0.018 according to t-test), indicating the ability of the sponge to produce DA from its precursor L-DOPA. Data are presented as the mean ± standard error of mean (SEM). *n* is the number of biologically independent sponge individuals. Each sample was collected separately from the field, processed independently, and analyzed in a single HPLC run.

It was observed that the DA content in the cell suspension did not differ from that of the intact sponge body (Supplementary Figure S4). Therefore, subsequent experiments were conducted using the intact sponge bodies, as they represent a more physiologically natural state of the organism. DA was detected in all examined sponge samples (Figure 1; Supplementary Figure S5). Initial analyses of sponges collected in the spring revealed DA levels close to the HPLC detection limit. Instead of pooling samples, we optimized the sample preparation method, which involved preconcentrating individual lysates using aluminum oxide (see Methods). This allowed us to reliably detect DA in individual samples and include all spring samples as independent biological replicates. To address the challenges posed by unequal group sizes and potential heteroscedasticity, we selected non-parametric statistical methods robust to these conditions. Post hoc pairwise comparisons using Dunn’s test were conducted following the Kruskal–Wallis test to identify significant differences between seasonal samples (χ^2^ = 8.3205, df = 2, p = 0.02; spring samples (*n* = 6) differed significantly from autumn samples (*n* = 3), p = 0.0221, and from winter samples (*n* = 3), p = 0.0234). The DA level (pM/mg of tissue) observed in spring samples was at one order of magnitude lower than the level in autumn and winter samples (Figure 1A). The NA levels were an order of magnitude lower than those of DA, and were similar in winter and spring samples (0.00624 and 0.00461 pM/mg tissue, respectively). DA and NA were undetectable in the seawater containing the sponges (Supplementary Figure S5).

In order to find out whether the sponges were able to produce DA from its precursor L-DOPA, we conducted an experiment in which sponges were incubated with 10 µM solution of L-DOPA. The results showed that DA levels increased following incubation with L-DOPA (Figure 1B). Similar results were obtained for cold-water sponge *S. ciliatum* (cl. Calcarea) (Supplementary Figure S6).

Thus, DA levels in *H. dujardini* varied across the studied life cycle periods, likely reflecting the sponge’s metabolic dynamics.

### *H. dujardini* enzymes for DA and NA synthesis

Genomic and transcriptomic data were leveraged to reconstruct the DA biosynthetic and metabolic pathways. DA is synthesized from tyrosine by the action of two enzymes: tyrosinase/tyrosine hydroxylase [EC:1.14.18.1/EC:1.14.16.2] and DOPA decarboxylase [EC:4.1.1.28]. A protein with homology to tyrosine hydroxylases was found in the genome of *H. dujardini* (XNM04295.1), although the reconstructed phylogeny showed it branching among a more inclusive group of phenylalanine hydroxylases, before the emergence of metazoan tyrosine and tryptophan hydroxylase groups, which are presumably specific to the bilaterian lineage (Supplementary Figure S7). Tyrosinases include 2 *H. dujardini* proteins (PQ658158 and PQ658159) and a cluster of proteins in *S. ciliatum* (XP_065187117.1 and related sequences) (Supplementary Figure S8). The majority of detected tyrosinases have similar domain architectures, with a secretory signal peptide, a copper binding site, and a transmembrane domain near the C-end (Supplementary Figure S9). The two tyrosinases from *H. dujardini*, however, lack detectable transmembrane regions while possessing a signal peptide, suggesting that they might be secretable. The enzyme required for the conversion of L-DOPA to DA–DOPA decarboxylase or Aromatic Amino Acid Decarboxylase (AADC) – was not found in *H. dujardini* or others sponges of cl. Demonpongia (Supplementary Figure S10). Several homologous decarboxylases found in *H. dujardini* belong to the group of pyridoxal-dependent decarboxylase domain-containing proteins and glutamate decarboxylases (Supplementary Figure S10).

We also performed a search for the enzyme dopamine beta-hydroxylase (DBH; EC 1.14.17.1), which catalyzes the synthesis of NA from DA (Supplementary Figure S11). Three candidate proteins with a catecholamine-binding DOMON domain were discovered by similarity search in *H. dujardini*. Only one of the detected *H. dujardini* proteins displayed full domain architecture similar to the *Homo sapiens* DBH, and contained the enzymatic copper type II ascorbate-dependent monooxygenase domains (PF01082 and PF03712). The other two detected proteins presented truncated architectures with only the N-terminal DOMON domain intact. No transmembrane regions were predicted for these proteins, yet all three possess a secretory signal peptide.

Thus, only two tyrosinase homologs and a DBH-like protein that could be implicated in the DA and NA synthesis were found in *H. dujardini*. The sponge lacks metazoan tyrosine hydroxylase, typically involved in the conversion of tyrosine to L-DOPA, or DOPA decarboxylase, which is central to the biosynthesis of catecholamines and trace amines. By contrast, *S. ciliatum* (cl. Calcarea), as well as several representatives of cl. Homoscleromorpha (*Corticium candelabrum*, *Oscarella lobularis*) were found to possess orthologs of DOPA decarboxylases.

### Analysis of the bacterial community, associated with *H. dujardini*

**FIGURE 2:**
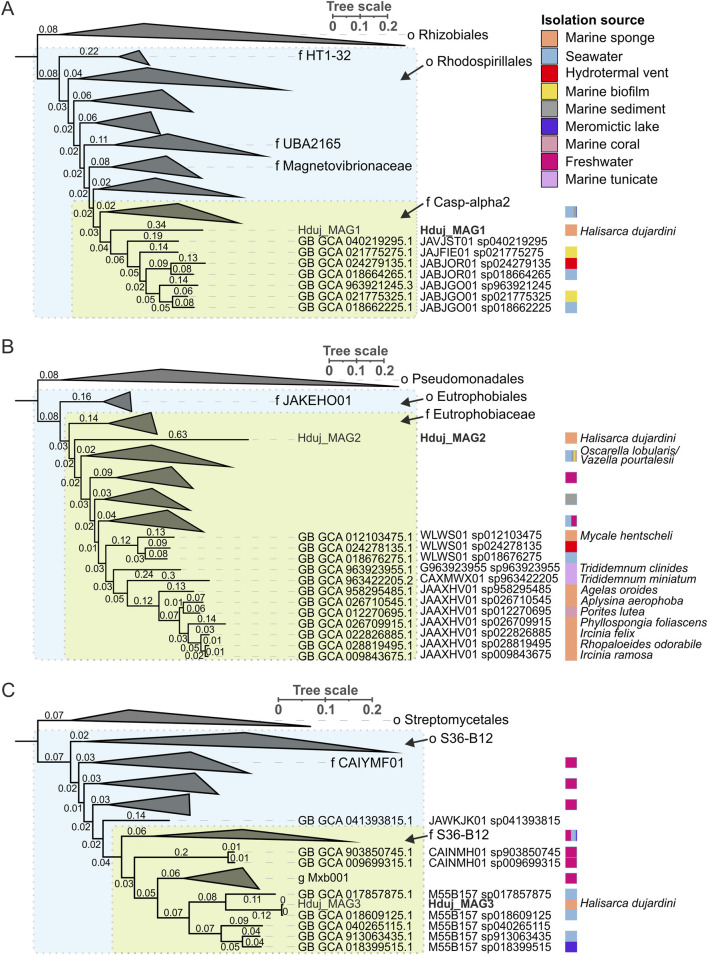
Phylogenetic analysis of bacterial species associated with *H. dujardini*. **(A)** Maximum-likelihood tree of o. Rhodospirillales. Rhizobiales order is shown as an outgroup. **(B)** Maximum-likelihood tree of o. Eutrophobiales. Pseudomonadales order is shown as an outgroup. **(C)** Maximum-likelihood tree of o. S36-B12. Streptomycetales order is shown as an outgroup. For all panels, phylogenetic distances are indicated on branches. The isolation source of MAGs is color-coded using a color strip. Color strip legend is shown in panel A. *H. dujardini*-associated MAGs are highlighted in bold. Phylogenetic trees were constructed using GTDB-Tk reconstruct (GTDB release 226) (https://doi.org/10.1093/nar/gkab776) (https://doi.org/10.1093/bioinformatics/btac672) and visualized with iTol v6 (Letunic and Bork, 2024).

Since the enzymes required for DA synthesis were not found in the *H. dujardini* genome, we decided to examine whether they could be present in its bacterial symbionts. In order to reconstruct genomes of sponge-associated bacteria, we generated three shotgun metagenome datasets (two short-read–Hal and Hal-pro datasets; and one long-read–the Nanopore dataset) for 2 *H. dujardini* samples and performed a cross-metagenome assembly followed by binning and refinement of metagenomic bins. This resulted in the reconstruction of three high-quality MAGs, which represent a novel genus within the Casp-alpha2 family (the Hduj_MAG1; Alphaproteobacteria/Rhodospirillales), a novel genus within the Eutrophobiaceae family (the Hduj_MAG2; Gammaproteobacteria/Eutrophobiaceae), and the M55B157_sp018609125 species (the Hduj_MAG3; Actinomycetes/S36-B12), based on the GTDB phylogeny reconstruction (Table 1). Further, we analyzed the ecology of related bacterial species to determine if they belong to bacterial taxa commonly associated with sponges. Other genomes from the Casp-alpha2 family were obtained from different marine environments (seawater, sediments, biofilms) and can be considered free living (Figure 2A). Similarly, M55B157_sp018399515 bacterium was previously isolated from seawater and other species of the same family S36-B12 were identified in seawater and freshwater environments (Figure 2C). Many of the previously obtained Eutrophobiaceae genomes (order Eutrophobiales) originated from marine invertebrates and were frequently found in marine sponges (Figure 2B). These observations indicate that the reconstructed MAGs are typical inhabitants of marine environments and that at least one of the MAGs is often associated with marine animals, such as sponges.

**TABLE 1:** Quality metrics and taxonomic annotation of *H. dujardini*-associated MAGs.

MAG ID	Completeness, contamination (%)	MAG taxonomy (GTDB)
Hduj_MAG1	95.12, 9.83	d__Bacteria; p__Pseudomonadota; c__Alphaproteobacteria; o__Rhodospirillales; f__Casp-alpha2; g__; s__
Hduj_MAG2	92.8, 0.01	d__Bacteria; p__Pseudomonadota; c__Gammaproteobacteria; o__Eutrophobiales; f__Eutrophobiaceae; g__; s__
Hduj_MAG3	96.14, 0.33	d__Bacteria; p__Actinomycetota; c__Actinomycetes; o__S36-B12; f__S36-B12; g__M55B157; s__M55B157 sp018609125

Enzymes for the biosynthesis of aromatic amino acids were found in all MAGs of *H. dujardini*-associated bacteria (Supplementary Tables S4, 6). Two genes for enzymes involved in the synthesis of serotonin (5-HT) and a gene with similarity to aromatic-L-amino-acid/L-tryptophan decarboxylase (K01593) were predicted in the Hduj_MAG3 (M55B157_sp018609125) MAG with BlastKOALA (Table 2). Although the reconstructed phylogeny of decarboxylases placed the discovered decarboxylase outside of the AADC clade (Supplementary Figure S10), its domain structure suggests that it can potentially synthesize DA from its precursor (Supplementary Figure S12). AADC was not found in two other reconstructed MAGs or any medium- and low-quality metagenomic bins obtained with binny. We then searched for the cofactor required for AADC, as well for other functions - vitamin B6 (pyridoxal 5′-phosphate) - that cannot be synthesized by animals. Enzymes involved in B6 biosynthesis were found in all three reconstructed MAGs (Supplementary Figure S13) with a complete pathway found in the Hduj_MAG3 (M55B157_sp018609125) MAG (Supplementary Table S5). No other enzymes involved in NA/A synthesis were found in the MAGs (Table 2).

**TABLE 2:** The occurrence of genes encoding enzymes involved in the synthesis of monoamines in MAGs of *H. dujardini*-associated bacteria. Tyr, tyrosine, DA, dopamine, NA, L-noradrenaline, A, L-adrenaline, MNP, L-metanephrine, TPH, tryptophan, TPA, tryptamine, 5-HT, serotonin, 5HTP, 5-hydroxy-L-tryptophan.

MAG ID	Tyr- > L-DOPA				L-DOPA- > DA		DA- > NA	NA- > A	A- > MNP	TPH- > TPA	TPA->5-HT	TPH- > HTrp	5HTP->5-HT
	K00501	K24287	K00505	K00422	K01592	K01593	K00503	K00553	K00545	K01593	K24541	K00502	K01593
Hduj_MAG1	-	-	-	-	-	-	-	-	-	-	-	-	-
Hduj_MAG2	-	-	-	-	-	-	-	-	-	-	-	-	-
Hduj_MAG3	-	-	-	-	-	+	-	-	-	+	-	-	+

The AADC gene was also found in *H. dujardini-*associated macroalga *Fucus serratus* (Supplementary Figure S10).

Thus, the actinomycetes M55B157_sp018609125, a putative sponge symbiont, and macroalga *Fucus serratus*, a common sponge substrate, might be potential sources of DA in *H. dujardini*.

Could synthesized DA influence sponge cells? To address this question, we searched for receptors capable of binding DA and investigated the potential for DA-mediated post-translational modifications of proteins. Additionally, we analyzed the differential expression of genes encoding proteins involved in DA metabolism across the sponge’s annual cycle and during dissociation–reaggregation events to assess their possible functional roles.

### G-protein coupled receptors

Protein domain searches identified six genes in the *H. dujardini* genome that belong to the Rhodopsin-like GPCR family. One of the identified GPCRs contained an array of leucine-rich repeats, and in the reconstructed phylogeny grouped with glycoprotein hormone receptors, which also have similar domain architectures (Supplementary Figure S14). Another receptor protein from *Halisarca* had a relatively divergent GPCR domain, and failed to cluster with any specific clade of GPCRs. The remaining four receptor proteins formed well-supported clades with uncharacterized GPCRs from other basal metazoans. Three of these receptors, GPCR 1 (PV768532), 2 (PV768533), and 3 (PV768534), cluster with a group of receptors from another sponge - *Amphimedon queenslandica* (Supplementary Figure S14). The fourth receptor (PV768535) falls outside of this sponge cluster and groups with a clade of GPCRs that include among others human eicosanoid receptors (Supplementary Figure S14). Further analyses were performed for the *Halisarca* proteins GPCR one to four to assess their expression in the sponge and potential to bind DA.

**FIGURE 3:**
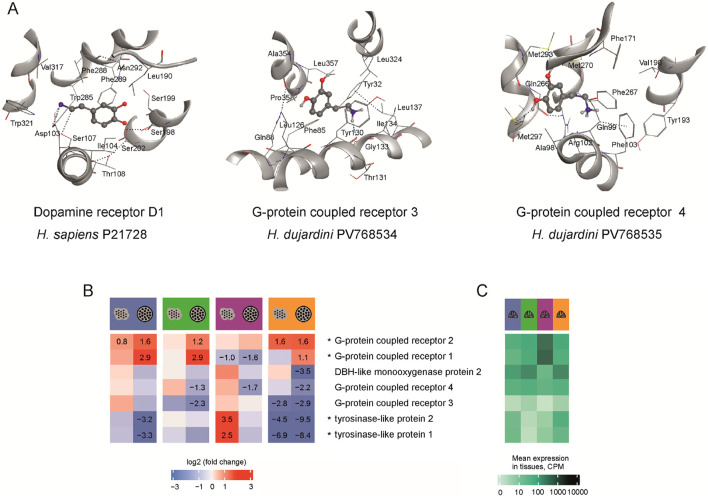
**(A)** The modeled structures of potential binding sites for 5-HT, NA and DA in the human DA receptor 1 (P21728), and G-protein coupled receptors 3 (PV768534) and 4 (PV768535) of *H. dujardini.* The amino acids are numbered according to the sequence of each protein. The hydrogen bonds are shown as dotted lines. M270 and M293 of receptor four form hydrogen bonds with the hydroxyl groups of dopamine. **(B)** Expression in CPM of *H. dujardini* G-protein coupled receptors, DBH-like and tyrosinase-like genes across reaggregation stages. The heatmap shows log fold changes in expression for dissociated cells (large dots) and aggregates (small dots within circles) compared to the intact sponges in corresponding seasons, with numerical values provided only for significant changes (p < 0.001). **(C)** Expression in intact sponges collected in various seasons (blue - winter, green - spring, lilac - summer, orange - autumn). The expression data is detailed in Supplementary Table S8.

To determine whether the identified receptors are capable of binding 5-HT, DA, and NA we performed docking analysis using MOE 2019.0102 (Molecular Operating Environment, 2019, Canada). For this purpose, we modeled the protein structures (GB AS) in MOE using homology modeling based on AlphaFold structures of the closest homologs (Supplementary Table S6), and invertebrates: fruit fly *Drosophila melanogaster* (cl. Insecta), the nematode *Caenorhabditis elegans* (cl. Chromadorea) and *Hydra vulagris* (cl. Hydrozoa) (see Supplementary Table S7). Binding sites for 5-HT, NA, and DA were identified within these structures. For all receptors, potential binding sites were found, formed by amino acid residues listed in Table 3 and Supplementary Table S7. The highest affinity for DA was predicted for *H. dujardini* G protein–coupled receptors 3 and 4, while the highest affinity for 5-HT was predicted for the *C. elegans* G protein–coupled receptor (Q86ME6) and *H. dujardini* G protein–coupled receptor 1 (Supplementary Table S7). Despite the variability of the amino acids involved in the binding, the general site structure of studied invertebrate G-protein coupled receptors is similar to the DA binding site in the human DA receptor D1 (Supplementary Table S7). The feature of G-protein coupled receptor four is the presence of two hydrogen bonds with methionine (Figure 3A). The closest homolog of receptor 3 in the tropical sponge *A. queenslandica* has a similar 3D structure and binding sites. It exhibits comparable affinity for NA, while its affinity for 5-HT and DA is lower than that of *H. dujardini* (see Supplementary Table S7; Supplementary Figure S15). The closest *A. queenslandica* homolog of receptor four lacks suitable binding sites for NA, DA, and 5-HT.

**TABLE 3:** The potential binding sites for 5-HT, NA and DA in G-protein coupled receptor 3 and four of *H. dujardini* and human DAD 1, predicted by MOE. The amino acids are numbered according to the sequence of each protein.

Name, GenBank AN	Binding site	Affinity to 5-HT binding, kkal/mol	Affinity to NA binding, kkal/mol	Affinity to DA binding, kkal/mol
G-protein coupled receptor 3 PV768534	Phe85 Gln88 Leu126 Ser129 Tyr130 Gly133 Ile134 Leu137 Tyr320 Leu324 Ala354 Leu357	−5.19756	−4.93133	−5.08519
G-protein coupled receptor 4 PV768535	Ala98 Gln99 Arg102 Phe103 Leu168 Phe169 Ser170 Phe171 Gln266 Phe267 Met270 Met293 Gly296 Met297	−4.85457	−5.82788	−5.71646
Human dopamine receptor D1 7LJD	Asp103 Ile104 Ser107 Thr108 Leu190 Ser198 Ser199 Ser202 Trp285 Phe288 Phe289 Asn292 Val317 Trp321	−5.17882967	−4.86117744	−4.98677

The annual life cycle of the Arctic sponge *H*. *dujardini* is closely tied to seasonal fluctuations in photoperiod, temperature, and the chemical composition of White Sea water. Key reproductive and developmental events are temporally segregated: spermatogenesis and oogenesis occur during the winter and extend into spring, larval release and metamorphosis take place in summer, and somatic growth peaks in autumn. Seasonal shifts in seawater chemistry, particularly, in bioavailable metal ions also influence sponge physiology. Notably, concentrations of copper and zinc, as well as pH, increased during the autumn–winter period (Supplementary Table S9). In contrast, DA levels in seawater remained stable low throughout the annual cycle (Figure 1). These seasonal metabolic shifts are reflected in the differential expression of proteins involved in DA turnover during cell reaggregation. To link these physiological transitions to underlying molecular regulation, we analyzed our previously published bulk RNA-Seq (BioProject PRJNA594150) and single-cell RNA-Seq (Zenodo: doi.org/10.5281/zenodo.14981466) datasets (see Supplementary Tables S2, 3). Because samples from each season were processed in separate sequencing batches, and full batch correction would risk removing biologically meaningful variation (see Methods), cross-season comparisons are interpreted qualitatively. Accordingly, our primary focus was on within-season contrasts during reaggregation (Figure 3B), using gene expression levels in intact sponge tissue as the baseline reference (Figure 3C).

Mechanical dissociation of the sponge into individual cells caused pronounced upregulation of G-protein coupled receptors 1 and 2, with expression further increasing in cell aggregates 24 h post-dissociation (Figure 3B). Their elevated baseline levels in summer intact bodies (Figure 3C) likely explain the exception observed for receptor 1, which showed downregulation during reaggregation in summer samples. In contrast, receptor 4 was consistently decreased in aggregates, and receptor 3 showed negative shifts in several contrasts, most notably in autumn and spring (Figure 3B). These bulk RNA-Seq patterns align with single-cell data, which reveal receptor 2 as broadly expressed across most cell clusters, whereas receptors 3 and 4 are confined to more restricted subsets (clusters 9/13 and 14, respectively; Supplementary Figure S16). Specifically, GPCR3 is predominantly expressed in pinacocyte-associated clusters (9 and 13), whereas GPCR4 is expressed in choanocytes undergoing transition toward a pinacocyte-like state (cluster 14). The limited cellular distribution of receptors 3 and 4 likely contributes to their variable responses to dissociation across seasons, whereas the widespread expression of receptor 4 underlies its stable upregulation during reaggregation. Tyrosinase genes were downregulated after dissociation and reaggregation, with the steepest decline observed in autumn, the period of active sponge body growth (Figure 3B). DBH-like monooxygenase two was likewise reduced in 24-h aggregates across all seasons, with its lowest baseline expression in summer samples (Figures 3B,C).

Thus, changes in the DA content in the sponge body during the annual cycle reflect the work of sponge DA turnover enzyme system connected with their micro- and macro-symbiotic environment.

### Analysis of post-translational modifications (PTMs) of *H. dujardini* proteins

Next, we investigated sponge protein modifications by monoamines. Our previous studies demonstrated that *H. dujardini* possesses transglutaminase-3 like and transglutaminase-5 like that could potentially mediate protein monoaminylation (Finoshin et al., 2024). We incubated native sponge protein lysates with MDC (monodansylcadaverine), a transglutaminase substrate, and subsequently separated the proteins by SDS-PAGE. MDC incorporation into sponge proteins was assessed by detecting its fluorescence. Strongest signal was observed in the 40–45 kDa region (Supplementary Figure S17), indicating monoamine incorporation and suggesting that transglutaminase-mediated protein modification may occur. Inhibition of transglutaminase activity with cystamine dihydrochloride reduced the MDC fluorescence by 92% (*p* < 0.029, *n* = 4) Supplementary Figure S17. The protein band corresponding to 40–45 kDa was selected for PTM analysis. MDC modification at glutamine 42 (Q42) and 50 (Q50) of the *H. dujardini* actin isoform HdA1/2/3 (accession QSX72278.1) was identified in this band (Supplementary Figure S18; Supplementary Table S10). Gel bands of the same molecular weight were excised from samples obtained from sponges collected with distinct periods of body growth and reproduction to investigate potential protein monoaminylation events. The number of samples and proteins that have been detected are shown in Supplementary Table S11.

**FIGURE 4:**
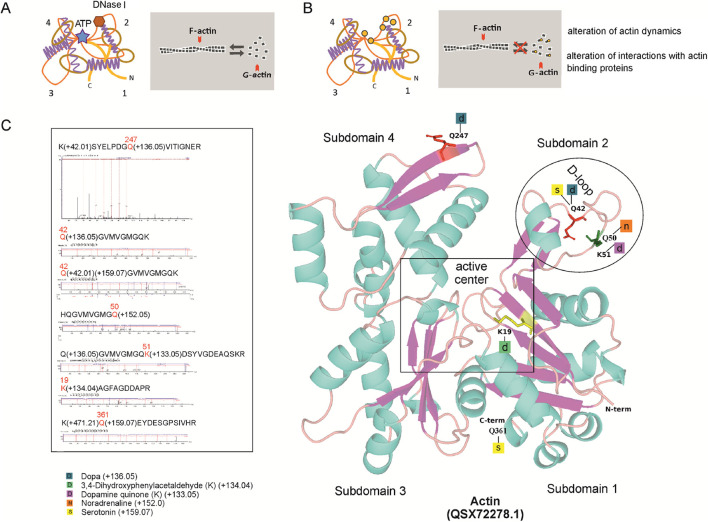
*H. dujardini* actin isoform HdA1/2/3 dopaminylation. **(A)** The changes in actin dynamics under normal conditions. **(B)** Post-translational modifications by DA, its derivatives and 5-HT, indicated by orange circles, can disturb actin dynamics. **(C)** Predicted 3D structures of *H. dujardini* HdA1/2/3 actin isoform (QSX72278.1), generated using the AlphaFold v3 web server, with putative post-translational modifications of amino acid residues (see Supplementary Tables S12, 13). Corresponding mass spectrometry fragmentation spectra for the modified peptides are also shown.

By using mass spectrometry, the peptides bearing DA, NA, and 5-HT resulting from enzymatic activity involving transglutaminases (PTMs on glutamine residues) were identified. In addition, non-enzymatic dopamine-related PTMs involving lysine (K) and cysteine (C) residues were analyzed (Figure 4; Supplementary Figure S19; Supplementary Tables S12, 13).

Notably, the modified peptides of *H. dujardini* HdA1/2/3 actin isoform (QSX72278.1) were found only in spring, summer, and autumn samples. The proportion of actin-derived peptides within the total peptide pool differs between the winter months (January–March) and the late spring/autumn months (May and November) (Supplementary Table S10). Statistical analysis revealed a highly significant difference between these periods. Using Pearson’s chi-squared test with Yates’ continuity correction, the proportion of actin peptides in January–March was significantly different from that in May–November (χ^2^ = 203.56, df = 1, p < 2.2 × 10^−16^). A broader comparison across all sampled months further confirmed this temporal variation: the relative abundance of actin peptides among the total peptide pool differed significantly by month (χ^2^ = 1,039.3, df = 5, p < 2.2 × 10^−16^). These results indicate that actin peptide representation varies in a season-dependent manner. The modified *H. dujardini* actin isoform HdA1/2/3 (QSX72278.1) amino acid residues involved in jasplakinolide, vinculin, DNase I, and ADP binding (K19, Q42, K51, Q247) (Figure 4) are found mainly in the sponge during the growth period (autumn samples). Given that mass spectrometry analysis covered thousands of proteins, monoaminylated peptides appear to be exceptionally rare in sponges. All the monoaminylated proteins that have been detected are associated with the cytoskeleton (Supplementary Tables S12, 13). In *H. dujardini*, 5-HT was found to covalently modify glutamine residues Q42 and Q361, and NA modified Q50, in the actin isoform HdA1/2/3. Additionally, NA was detected on Q4 of twinfilin (Supplementary Table S13). A dopaminylated peptide with Q103 in gelsolin-like protein one was found in *S. ciliatum* (Supplementary Figure S20).

**FIGURE 5:**
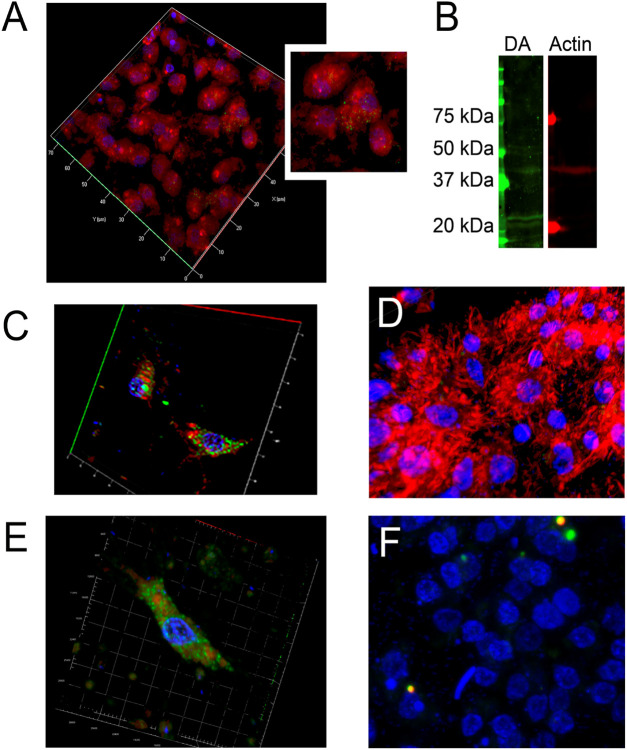
Immune fluorescence of DA in *H. dujardini* sponge cells. **(A)** The representative samples of 3D projection of a confocal image of β-Actin (red, stained with polyclonal rabbit anti-β-Actin antibody) and Glu-Lys link (green, detected using monoclonal mouse anti-Glu-Lys link antibody) staining in sponge body cells (*n* = 3). The region containing Glu-Lys-positive cells is highlighted. **(B)** Verification of antibody specificity determined by the Western blot of sponge body lysates. Dopaminylated peptides were detected as distinct, narrow bands using a polyclonal rabbit anti-dopamine (anti-DA) antibody (green), while β-actin was detected as single band with a monoclonal mouse anti–β-actin antibody (red) (*n* = 3). **(C,E)** The representative samples of 3D projections of confocal images displaying representative cells co-stained for DA (green) and β-Actin (red) (*n* = 4). **(D)** 3D projection of confocal image of mature sponge body cells showing DA (green) and F-actin labeled with phalloidin (red). Fluorescence detection was performed using Alexa Fluor 546-conjugated goat anti-rabbit IgG or Alexa Fluor 633-conjugated donkey anti-mouse IgG secondary antibodies. DA-stained proteins detected as small immunoreactive dots in the cytoplasm of sponge cells are shown. **(F)** The representative sample of 3D projection of confocal image of sponge body cells, without primarily antibodies to DA and F-actin (*n* = 3). Scale bar: 10 μm.

The immunofluorescent staining using specific antibodies showed the presence of DA within actin in cytoplasm of the *H. dujardini* body cells, collected in autumn (Figures 5A–F). The Glu-Lys crosslink, mediated by transglutaminase, was detected as small immunoreactive dots both in the cytoplasm and nucleus of *H. dujardini* sponge cells (Figure 5A). DA immunoreactivity appeared as clusters of varying-sized dots both large and small mainly in certain cells of mature sponge (Figures 5C,E).

Therefore, our findings indicate that monoamines primarily modify cytoskeleton proteins in the examined sponges. In *H. dujardini*, actin undergoes dopaminylation in certain cells at the period of sponge growth.

## Discussion

**FIGURE 6:**
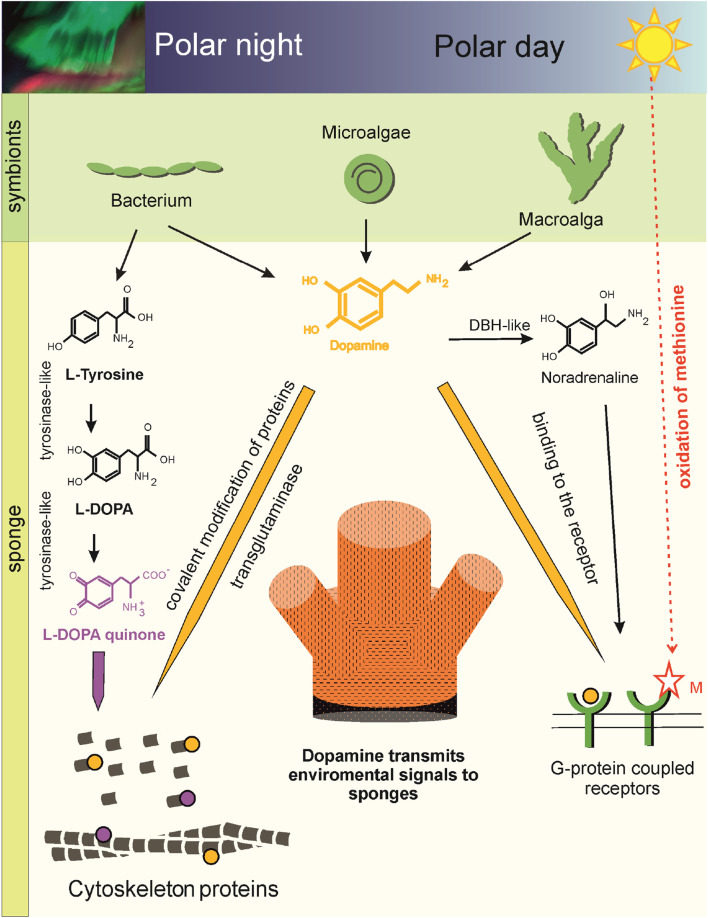
Scheme of DA-mediated cellular plasticity in the Arctic sponge *H. dujardini* (cl. Demospongia). Aromatic amino acids, including tyrosine, a precursor of DA, are synthesized by the bacterial symbionts of the sponges (Metagenomic data). Tyrosine converted to DA in the bacterial symbiont of the sponge *H. dujardini* (Table 2), micro- and macroalgae can be delivered to the sponge, because genes encoding enzyme required for the conversion of L-DOPA to DA - DOPA decarboxylase (AADC) is not found in cl. Demospongia (Supplementary Table S10) (Metagenomic and transcriptomic data, HPLC). DA can influence the functioning of *H. dujardini* cells through binding to G protein-coupled receptors. The sponge has several potentially functional receptors (Supplementary Figure S14) (Genome and Sc RNA seq data). The presence of two hydrogen bonds with methionine in the *H. dujardini* G protein-coupled receptor suggests its dependence on the redox status of the environment and predicts a higher affinity for DA (Molecular docking). In spring, daylight hours increase in polar latitudes. DA levels in sponges decrease, and the redox-sensitive G protein-coupled receptor becomes more sensitive to solar light. This period coincides with structural changes in the aquifer system of the Arctic sponge *H. dujardini*, connected with gametogenesis. DBH-like and G protein-coupled receptors showed dynamic expression patterns during morphogenetic transitions in the sponge *H. dujardini* (RNA seq data). DA signaling is also supported by posttranslational dopaminylation of cytoskeletal proteins. Transglutaminases are thought to modify glutamine residues in *H. dujardini* actin (LC-MS/MS data). The modifications may disrupt actin polymerization, the ATPase cycle, and interactions with binding partners, leading to oxidative stress. DAQ modification of Lys forms hydrogen bonds or metal coordination complexes. DA modified actin peptides were not detected in winter sponge samples, when the cold-water sponge *H. dujardini* begins a new reproductive cycle and undergoes spermatogenesis and oogenesis. Fluctuations in cellular DA levels and actin dopaminylation during the sponge life cycle suggest the ancient role of DA in modulating cellular plasticity through both transcriptional and post-translational regulatory mechanisms connecting within environment.

Sponges diverged from other animal groups over 600 million years ago and retain features common to the ancestors of all metazoans. They are composed of diverse cell populations (Sogabe et al., 2019) capable of specialized functions and interactions, including close associations with microbes, micro- and macroalgae, indicating the existence of molecular prerequisites for signaling in response to external factors. Despite the absence of a classical nervous system, sponge cell types express genes associated with neuronal signaling (Musser et al., 2021). The diversity of sponge classes and their symbiotic communities suggests that molecular components of signaling have co-evolved in a habitat-dependent manner. Genes of catecholamine biosynthesis are not fully represented in the genomes of sponges of cl. Demospongia and their symbionts, but are present in sponges of cl. Calcarea (*S. ciliatum*) and cl. Homoscleromorpha (*Corticium candelabrum*, *Oscarella lobularis*) (Supplementary Figure S10). The L-DOPA in some Demospongia can be produced by tyrosinases or TH of bacterial symbionts (Xiang et al., 2023). The integration of HPLC data, sponge genomics, metagenomic reconstruction of symbiotic microbiota, and identification of monoaminylated proteins in this study reveals that DA in the cold-water demosponge *H. dujardini* is far more than a biochemical curiosity. We hypothesize that it functions as a signaling molecule that is dynamically modulated across the Arctic annual cycle to regulate fundamental physiological, structural, and developmental processes in direct response to environmental cues such as photoperiod, temperature, metal ion availability, and microbial metabolic rhythms (Figure 6).

Prior effect size estimates were unavailable due to the novelty of this work, since no comparable seasonal studies of DA dynamics in wild sponges had been published. Given the logistical and ethical constraints of working with wild Arctic sponge populations, where sample availability is inherently limited and seasonal access is restricted, the analytical approach employed represents the most rigorous and appropriate use of the available biological material. To address the challenges posed by unequal group sizes and potential heteroscedasticity, non-parametric statistical methods robust to these conditions were selected.

DA concentrations in sponge body tissue are not static and undergo statistically significant oscillations, peaking in autumn and winter and decreasing by an order of magnitude in spring (Figure 1A). Critically, DA is undetectable in the surrounding seawater, confirming that these fluctuations reflect tightly regulated endogenous turnover rather than passive environmental uptake. This seasonal rhythm is not mirrored in NA, whose levels remain consistently low, suggesting that DA and NA serve non-redundant physiological roles.

This biochemical rhythm is mechanistically explained by a striking genomic deficiency since the sponge *H. dujardini* lacks TH and AADC, the canonical metazoan enzymes required for *de novo* DA biosynthesis from tyrosine, indicating it cannot autonomously produce DA. However, metagenomic analysis of its microbiome reveals that this can be compensated by symbiotic bacteria. Notably, the actinomycete *M55B157_sp018609125* encodes aromatic L-amino acid decarboxylase (AADC; K01593), which is potentially capable of converting L-DOPA to DA and 5-HTP to 5-HT (Table 2). Additionally, the macroalga *Fucus serratus*, commonly associated with the sponge, also possesses AADC, suggesting a multi-partner, cross-kingdom metabolic consortium that supplies either DA precursors or the final product to the host. It is interesting that in the central nervous system of mammals the non-dopaminergic neurons lacking some of the enzymes for DA synthesis demonstrate cooperated synthesis of DA (Kozina et al., 2017). The bacterial MAGs, associated with the cold-water sea sponge *H. dujardini*, encode biosynthetic pathways of aromatic amino acids, vitamins, and cofactors (including thiamine, riboflavin, tetrahydrofolate, cobalamin, pyridoxal, and molybdenum cofactor), indicating a potential supplementation of the host by its symbionts with these vital compounds (Supplementary Tables S4, 5). Amino acids and 5-HT can be synthesized by sponge-associated symbiotic bacteria (Waterworth et al., 2021; Leys, 2015). Exogenous L-DOPA treatment nearly doubled endogenous DA levels, functionally validating this model: the sponge holobiont possesses the enzymatic machinery, likely microbial, to complete DA synthesis, but its activity is gated by precursor availability and seasonal symbiont dynamics. In *S. ciliatum*, which retains AADC, L-DOPA treatment induced a higher DA increase than in *H. dujardini*, consistent with host-dependent enzymatic capacity. The seasonal expression patterns of key genes underscore active regulation of DA signaling (Figures 3B,C). Tyrosinase is upregulated in autumn and coincides with DA peaks and periods of tissue growth, while the specific cell cluster expressing G-protein coupled receptor four is elevated during the spring (Lyupina et al., 2025). This receptor contains a redox-sensitive methionine residue and is predicted *in silico* to have high affinity for DA, suggesting it may act as a light- or redox-responsive sensor when the DA levels are low. In Arctic spring, the end of polar night and increasing daylight coincide with the remodeling of sponge aquiferous system, associated with gametogenesis (Ereskovsky, 2000). In *A. queenslandica* larvae, which has been exposed to agonists and antagonists of bilaterian DA and trace amine-associated receptors, marked disturbances in larval negative phototactic swimming behavior were noticed (Xiang et al., 2023). The synthesis of DA, as well as its release and signaling in the retina, olfactory bulb, ventral tegmental area, and striatum, are orchestrated in a circadian manner in mammals (Mendoza and Challet, 2014). DA may also interact with photosynthetic symbionts: biological polydopamine can act as an electron-coupling matrix, enhancing light-harvesting via photosynthetic pigments on conductive surfaces (Labarile et al., 2024). The demosponge *Neopetrosia exigua* (Palau) with symbiotic cyanobacteria produced DA for anti-predator and anti-biofouling defense (Liu et al., 2004). Huge amounts of DA are secreted by the cells of the ciliate *Tetrahymena pyriformis* into the growth medium (Gundersen and Thompson, 1985), and DA is also used by alga *Ulvaria obscura* (Van Alstyne et al., 2006) for defense against foreign inhabitants of the communities.

Tyrosinase may generate L-DOPA for DA synthesis and cross-linking extracellular matrix proteins via DOPA-quinone formation to reinforce structural integrity during growth phases. The DOPA quinones undergo cross-linking reactions with Lys/His of neighboring proteins and form a sclerotized matrix structure (Cordingley, 1987; Smyth and Halton, 1983). Tyrosinase genes have not been found in the marine sponge *A. queenslandica*, *Trichoplax adhaerens* (Placozoa) and *Nematostella vectensis* (Cnidaria). It is possible that other enzymes such as peroxiredoxins and catechol oxidases may perform similar functions to tyrosinases in these animals. DBH-like monooxygenases involved in NA formation are present in all sponges. The sequences of two short genes, DBH-like monooxygenase protein one and DBH-like monooxygenase protein 3, of *H. dujardini* are terminated after the DOMON domain, i.e., these truncated homologs likely diverged relatively recently from the full-fledged DBH-like monooxygenase copy. In the tree, both short proteins group with the true DBH-like monooxygenase protein two but with different support (Supplementary Figure S11). NA is being explored as an antifouling agent due to its ability to deter the settlement of fouling organisms, but leaching of NA or its derivatives from coated surfaces into the surrounding water can expose non-target species like zooplankton. The *H. dujardini* gene expression of DBH-like monooxygenase protein 2 decreases in multicellular aggregates upon reaggregation and suggest that it may be involved in the regeneration processes (Figure 3B). Of particular interest is the differential expression of genes involved in DA turnover, which correlates with seasonal shifts in the sponge’s reproductive cycle as well as with dissociation and reaggregation events. It is known, that DBH-like regulates metamorphosis of *Sinonovacula constricta* (Li et al., 2020). Notably, the genes encoding GPCRs 3 and four are expressed in distinct cell clusters (Supplementary Figure S16). The proportion of cells expressing redox-sensitive GPCR 4 rises during spring, whereas GPCR 3 expressing cells are primarily observed within cellular aggregates (Lyupina et al., 2025). This pattern suggests that these GPCRs may serve distinct functional roles and that the cells expressing them likely exhibit different metabolic states. The molecular docking showed differences in the sensitivity of these receptors to monoamines (Supplementary Table S7). The interactions and binding energies we obtained are consistent with existing experimental data, supporting the plausibility of our model. The proposed binding sites for DA, NA and 5-HT appear structurally reasonable but require experimental verification. Future work using purified sponge receptor binding assays or site-directed mutagenesis, such as Met to Ala substitutions followed by functional characterization in yeast or other model systems, will be essential to transform these computational predictions into confirmed biochemical mechanisms. GPCRs play a key role in the perception of environmental signals and cellular communication in representatives of all branches of life. GPCRs of unicellular organisms are involved in chemotaxis and cell adhesion (Luo et al., 2023). In the social amoeba *Dictyostelium discoideum*, GPCRs play key roles in cell differentiation and multicellular morphogenesis (Pergolizzi et al., 2017; Kamimura and Ueda, 2021). In animals, the number of GPCRs increased dramatically, especially for the Rhodopsin-like family of GPCRs. The sponge *A. queenslandica* has about 100 Rhodopsin receptors, which form a separate lineage, and *Oscarella* has around 50. These proteins were part of the receptor repertoire of ancestral lineages, and appear to have been lost or expanded in each lineage independently. The genes implicated in the synthesis of monoamine neurotransmitters, such as phenylalanine hydroxylase, dopa decarboxylase and DBH-like genes and GPCRs were identified in the genomes of placozoans and ctenophores (Jin et al., 2024). Trichoplax (Placozoa) GPCRs can bind neurotransmitters (Jin et al., 2024). It has been shown that DA slows down the speed of movement, suppresses swimming in water and induces crawling on hard substrate in mollusks, and reduces locomotor rhythms in lampreys, crabs, and *Danio rerio* (Sawin et al., 2000; Vidal-Gadea and Pierce-Shimomura, 2012) through GPCR. The D1 receptors regulate the frequency and amplitude of body bending during movement of *C. elegans* in environments with different viscosity (Vidal-Gadea and Pierce-Shimomura, 2012) and also inhibit the basal slowing response in nematodes (Chase and Koelle, 2004; Lanson and Pandey, 2012; Vidal-Gadea and Pierce-Shimomura, 2012).

At the proteomic level, mass spectrometry and immunofluorescence confirm that DA is not merely a soluble signal but can covalently incorporated into structural proteins, primarily actin, via both enzymatic (transglutaminase-mediated glutamine modification) and non-enzymatic (lysine-DAQ adduct) pathways. These dopaminylation events target residues can be critical for actin function since K19, Q42, K51, Q247 residues impacting polymerization, ATP hydrolysis, and protein interactions. Notably, monoaminylated actin peptides were absent in winter samples, coinciding with the onset of the reproductive cycle (spermatogenesis and oogenesis), but were detected in cytoskeletal proteins during the somatic growth phase, which marks the final stage of the sponge annual cycle. Actin dynamics in sponges vary seasonally, with the highest abundance of both total actin peptides and monoaminylated peptides observed in autumn, corresponding to the period of active body growth. Importantly, the detection of DA-modifications on actin does not correlate with overall peptide abundance, and indicating that this modification is rare. In addition to dopaminylation, we also identified NA and 5-HT modifications on cytoskeletal proteins: specifically, on actin and twinfilin in *H. dujardini*, and on gelsolin in *S. ciliatum*. We propose that these PTM may selectively target specific cytoskeletal proteins in sponge somatic cells at the end of the annual cycle. Given that *H. dujardini* cells exhibit heterogeneous redox states and variable ferric ion/ferritin content (Adameyko et al., 2021; Finoshin et al., 2024; Lyupina et al., 2025) dopaminylation of proteins can be facilitated for cells with high ROS levels. This phenomenon paralleled in mammalian macrophages, where DA regulates iron homeostasis (Dichtl et al., 2018). Since the modified residues (e.g., Q42 in actin) are located near the nucleation and polymerization interface, such modifications could disrupt actin filament dynamics and serve as a signal for targeted protein turnover or stabilization. This seasonal “preparation” may facilitate cytoskeletal remodeling in anticipation of the next annual cycle. Interestingly, that serotonylation of actin and small GTPases regulates vascular tone and platelet function (Watts et al., 2009), while aberrant dopaminylation of proteins has been associated with oxidative stress, proteostatic dysfunction, and neurodegeneration in aging (Plotegher et al., 2017; Reddy et al., 2024). These findings suggest that while monoaminylation serve physiological regulatory roles, its dysregulation may contribute to age-dependent decline in cellular homeostasis. In sponges, among the earliest-branching metazoans, actin dopaminylation could reflect an ancient, evolutionarily conserved mechanism for modulating cellular plasticity. Thus, DA functions not only as a ligand for membrane receptors but also as a direct biochemical modulator of the cytoskeleton, translating environmental and metabolic states into structural outcomes. Future studies that track symbiont gene expression across seasons and assess how DA levels influence *H. dujardini* symbiont community composition will be essential for elucidating the biochemical basis of host-microbe and environment interactions in greater detail.

## Conclusion

The DA is present in sponges even when their own genomes lack a complete biosynthetic pathway, with the production likely delegated to a cross-kingdom symbiotic consortium. The seasonal dynamics of DA levels, receptor expression, and protein dopaminylation in the Arctic sponge *H. dujardini* reveal a sophisticated, environmentally tuned signaling system. Far from being a vestigial molecule, DA acts as an evolutionarily entrenched regulator that integrates photoperiod, microbial metabolism, redox state, and structural plasticity, contending for a crucial role in the ancestral mechanisms of cell–environment communication long before the emergence of a nervous system.

## Data Availability

The datasets presented in this study can be found in online repositories. The names of the repository/repositories and accession number(s) can be found in the article/Supplementary Material. Table S8 - Accession numbers in GenBank for mRNA sequences of H. dujardini proteins and expression data. Sequencing data are available under NCBI BioProject accessions PRJNA594150 (bulk RNA-seq) and Zenodo: doi.org/10.5281/zenodo.14981466 (WGS, scRNA-seq), PRJNA1347440 (metagenome). Genome data are available in Zenodo: doi.org/10.5281/zenodo.14981466. The mass spectrometry proteomics data have been deposited to the ProteomeXchange Consortium via the PRIDE (Perez-Riverol et al., 2025) partner repository with the dataset identifier PXD066419.
