# Supplementary material for: Evolutionary conservation of dopamine-mediated cellular plasticity in Arctic sponges (Porifera)
Source: Front Mol Biosci. 2025 Nov 17;12:1671771. doi: 10.3389/fmolb.2025.1671771 (PMC12665527; doi:10.3389/fmolb.2025.1671771)
Supplement: Supplementary file 3 [file Table1.docx]

Table S1. The PTMs of glutamine (Q), сysteine (C), and lysine (K) involving dopamine.

| Item | Modification | Released elements | Added Mass | Modified residue | Reference |
| --- | --- | --- | --- | --- | --- |
| 1 | + DA  Dopamine | -NH3  + C8 H11 NO2 | 136.05 | Glutamine (Q) | Farrelly, L.A., Thompson, R.E., Zhao, S. et al. Histone serotonylation is a permissive modification that enhances TFIID binding to H3K4me3. Nature 567, 535–539 (2019).  Zhang N, Gao S, Peng H, Wu J, Li H, Gibson C, Wu S, Zhu J, Zheng Q. [Chemical Proteomic Profiling of Protein Dopaminylation in Colorectal Cancer Cells.](https://pubmed.ncbi.nlm.nih.gov/38838187/)  J Proteome Res. 2024 Jul 5;23(7):2651-2660. doi: 10.1021/acs.jproteome.4c00379 |
| 2 | + DAQ  dopamine quinone | /  +[C8H9NO2](https://pubchem.ncbi.nlm.nih.gov/#query=C8H9NO2) | 151.06 | Cysteine (C) | Whitehead, R.E., Ferrer, J.V., Javitch, J.A. and Justice, J.B. (2001), Reaction of oxidized dopamine with endogenous cysteine residues in the human dopamine transporter. Journal of Neurochemistry, 76: 1242-1251. |
| 3 | + DA  Dopamine | /  +C8 H11 NO2 | 153.18 | Cysteine (C) | Zhang N, Gao S, Peng H, Wu J, Li H, Gibson C, Wu S, Zhu J, Zheng Q. [Chemical Proteomic Profiling of Protein Dopaminylation in Colorectal Cancer Cells.](https://pubmed.ncbi.nlm.nih.gov/38838187/)  J Proteome Res. 2024 Jul 5;23(7):2651-2660. doi: 10.1021/acs.jproteome.4c00379 |
| 3 | + DAQ  dopamine quinone | -H2O  +[C8H9NO2](https://pubchem.ncbi.nlm.nih.gov/#query=C8H9NO2) | 133.05 | Lysine (K) | Cristian Follmer, Eduardo Coelho-Cerqueira, Danilo Y. Yatabe-Franco, Gabriel D. T. Araujo, Anderson S. Pinheiro, Gilberto B. Domont, and David Eliezer Oligomerization and Membrane-binding Properties of  Covalent Adducts Formed by the Interaction of  -Synuclein with the Toxic Dopamine Metabolite  3,4-Dihydroxyphenylacetaldehyde (DOPAL) THE JOURNAL OF BIOLOGICAL CHEMISTRY VOL. 290, NO. 46, pp. 27660 –27679 |
| 4 | + DOPAL  3,4-Dihydroxyphenylacetaldehyde | -H2O  + C8H8O3 | 134.04 |  |  |
| 5 | + DC  Dopaminochrome | -H2O  +[C8H7NO2](https://pubchem.ncbi.nlm.nih.gov/#query=C8H7NO2) | 131.04 |  |  |
