# Supplementary material for: Evolutionary conservation of dopamine-mediated cellular plasticity in Arctic sponges (Porifera)
Source: Front Mol Biosci. 2025 Nov 17;12:1671771. doi: 10.3389/fmolb.2025.1671771 (PMC12665527; doi:10.3389/fmolb.2025.1671771)
Supplement: Supplementary file 4 [file Table12.docx]

**Table S12. Extended mass-spectrometry data for *H. dujardini* actin (QSX72278.1).**

Dopamylated peptides are shown for each sample.

| Description | Peptide | PTM |
| --- | --- | --- |
| Larva, June | **Q**(+136.05)GVM(+15.99)VGMGQK | Dopa; Q42 |
| Adult, May | **Q**(+136.05)GVMVGMGQK  **K**(+134.04)AGFAGDDAPR  Q(+136.05)GVMVGMGQ**K**(+133.05)DSYVGDEAQSKR | Dopa; Q42  3,4-Dihydroxyphenylacetaldehyde; K19  DAQ; K51 |
| Adult, August | K(+42.01)SYELPDG**Q**(+136.05)VITIGNER | Dopa; Q247 |
| Adult, September | **Q**(+136.05)GVMVGMGQK | Dopa; Q42 |
| Adult, November | **Q**(+136.05)GVMVGMGQKDSYVGDEAQSK | Dopa; Q42 |

**Table S13. Extended mass-spectrometry data for *H. dujardini* actin (QSX72278.1) and twinfilin-1a (PV611706 ).**

| Description | Peptide | Protein | PTM |
| --- | --- | --- | --- |
| Larva tissue, June | **Q**(+42.01)(+159.07)GVMVGMGQK  HQGVMVGMG**Q**(+152.05) | actin  actin | Serotonin; Q42  Noradrenaline; Q50 |
| Adult tissue, February | **Q**(+152.05)T(+114.04)GIEADAELKK | twinfilin-1a | Noradrenaline; Q4 |
| Adult tissue, September | K(+471.21)**Q**(+159.07)EYDESGPSIVHR | actin | Serotonin; Q361 |
| Adult tissue, November | K(+471.21)**Q**(+159.07)EYDESGPSIVHR | actin | Serotonin; Q361 |
