# Supplementary material for: Evolutionary conservation of dopamine-mediated cellular plasticity in Arctic sponges (Porifera)
Source: Front Mol Biosci. 2025 Nov 17;12:1671771. doi: 10.3389/fmolb.2025.1671771 (PMC12665527; doi:10.3389/fmolb.2025.1671771)
Supplement: Supplementary file 5 [file Table7.docx]

# Table S7. The 5-HT, NA and DA potential binding sites in G-protein coupled receptors 1-4 of *H. dujardini*, Beta-2 adrenergic receptor *Hydra vulgaris*, dopamine receptor 1 *Drosophila melanogaster*, dopamine receptor 1 *Caenorhabditis elegans*, galanin receptor type 3-like of *Amphimedon queenslandica*, and human dopamine receptor 1, predicted in MOE. The amino acids are numbered according to the sequence of each protein.

| Genbank AS, organism | Binding site | Affinity to  5-HT binding, kkal/mol | Affinity to  NA binding, kkal/mol | Affinity to DA binding, kkal/mol |
| --- | --- | --- | --- | --- |
| G-protein coupled receptor 1 PV768532, *H. dujardini* | Glu190  Leu193  Leu197  Trp200  Tyr206  Phe209  Phe210  Thr288  Ala289  Ser292  Ile293  Thr297  Phe305 | -5.20909 | -5.15248 | -5.02873 |
| G-protein coupled receptor 2 PV768533, *H. dujardini* | Val155  Asp156  Leu223  Trp226  Leu240  Leu241  Leu244  Leu245  Leu329  Phe330  Tyr333  Ile334 | -4.86228 | -5.04607 | -5.04777 |
| G-protein coupled receptor 3 PV768534, *H. dujardini* | \| Phe85 \| \| --- \| \| Gln88 \| \| Leu126 \| \| Ser129 \| \| Tyr130 \| \| Gly133 \| \| Ile134 \| \| Leu137 \| \| Tyr320 \| \| Leu324 \| \| Ala354 \| \| Leu357 \| | -5.19756 | -4.93133 | -5.08519 |
| G-protein coupled receptor 4 PV768535, *H. dujardini* | Ala98  Gln99  Arg102  Phe103  Leu168  Phe169  Ser170  Phe171  Gln266  Phe267  Met270  Met293  Gly296  Met297 | -4.85457 | -5.82788 | -5.71646 |
| XP_011404757  *Amphimedon queenslandica* | Tyr69  Ile102  Tyr170  Ser172  Val174  Ser250  Tyr267  Asn270  Met271  Phe274  Gln275 | -5,08175 | -4,99482 | -4,78382 |
| T2MGT0  *Hydra vulgaris* | Ile69  Ile72  Asn73  Arg76  Arg81  Phe83  Ser93  Leu94  Glu97  Lys177  Cys178  Asn179  Tyr266  Thr285  Glu286 | -5.1800661 | -4.5998907 | -4.4349465 |
| P41596  *Drosophila melanogaster* | Asn203  Asp204  Trp219  Asp223  Leu290  Phe292  Cys302  Ala303  Leu304  Asp305  Leu306  Phe409  Ala416  Lys420  Gly425  Phe428  Lys429  Thr432  Tyr436 | -5.0415 | -4.44902 | -4.33644 |
| Q86ME6  *Caenorhabditis elegans* | Asn66  Asp67  Trp82  Ile83  Asp86  Ile87  Cys161  Glu162  Met163  Arg164  Leu165  Pro166  Phe343  Asn347  Arg350  Ala351  Pro354  Ile362  Met363  Thr366  Tyr370 | -5.2489295 | -4.60544 | -4.68472 |
| 7LJD, *Homo sapiens* | Asp103  Ile104  Ser107  Thr108  Leu190  Ser198  Ser199  Ser202  Trp285  Phe288  Phe289  Asn292  Val317  Trp321 | -5.17882967 | -4.86117744 | -4.98677 |
