# Supplementary material for: Evolutionary conservation of dopamine-mediated cellular plasticity in Arctic sponges (Porifera)
Source: Front Mol Biosci. 2025 Nov 17;12:1671771. doi: 10.3389/fmolb.2025.1671771 (PMC12665527; doi:10.3389/fmolb.2025.1671771)
Supplement: Supplementary file 6 [file Table9.docx]

Table S9. The chemical content of White Sea water in different seasons. The content of all elements was determined using atomic emission spectrometry with inductive associated plasma, μg/l. The content of anions was determined by chromatography and capillary electrophoresis, μg/l.

| Parameter | May 2024 | July 2024 | December 2024 |
| --- | --- | --- | --- |
| pH | 7.76 | 7.74 | 6.8 |
| Ammonium | 0.24 | 0.11 | 0.19 |
| Iron | <0.1 | <0.1 | <0.1 |
| Potassium | 329.35 | 271.77 | 305.99 |
| Magnesium | 930.17 | 783.79 | 841.88 |
| Manganese | <0.01 | <0.01 | <0.01 |
| Calcium | 274.6 | 232.69 | 252.16 |
| Aluminum | <0.1 | <0.1 | <0.1 |
| Sodium | 7898.2 | 6676.53 | 7155.27 |
| Copper | <0.01 | <0.01 | 0.0363 |
| Plumbum | <0.01 | <0.01 | <0.01 |
| Arsenic | <0.05 | <0.05 | <0.05 |
| Cadmium | <0.001 | <0.001 | <0.001 |
| Zinc | 0.06 | 0.09 | 0.21 |
| Strontium | 5.33 | 4.57 | 4.94 |
| Hydrocarbonates | 137.86 | 111.63 | 129.32 |
| Carbonates | 0 | 0 | 0 |
| Fluorides | < 1 | < 1 | < 1 |
| Chlorides | 15968.1 | 14064.37 | 14835.76 |
| Nitrates | 3.87 | <1 | <1 |
| Sulfates | 1988.55 | 1713.35 | 1807.99 |
