# Supplementary material for: Evolutionary conservation of dopamine-mediated cellular plasticity in Arctic sponges (Porifera)
Source: Front Mol Biosci. 2025 Nov 17;12:1671771. doi: 10.3389/fmolb.2025.1671771 (PMC12665527; doi:10.3389/fmolb.2025.1671771)
Supplement: Supplementary file 7 [file DataSheet1.pdf]

Figure S1. *Halisarca dujardini* (A) on the substrate (*Fucus*) and *Sycon ciliatum* (B). Sample collection area in different seasons of the year: Winter (C, D), Spring (E), and Autumn (F, G)

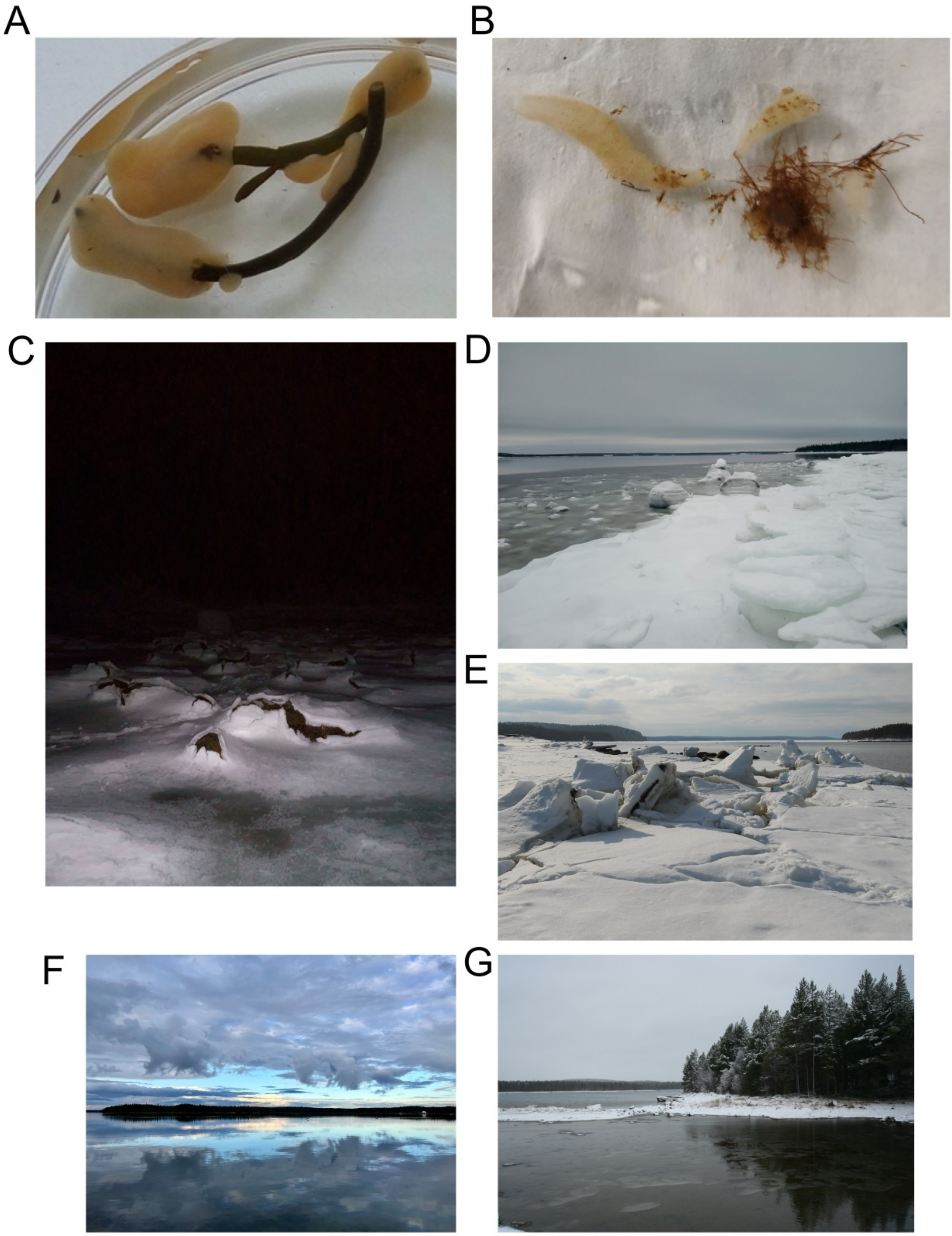

Figure S2. PCA batch correction

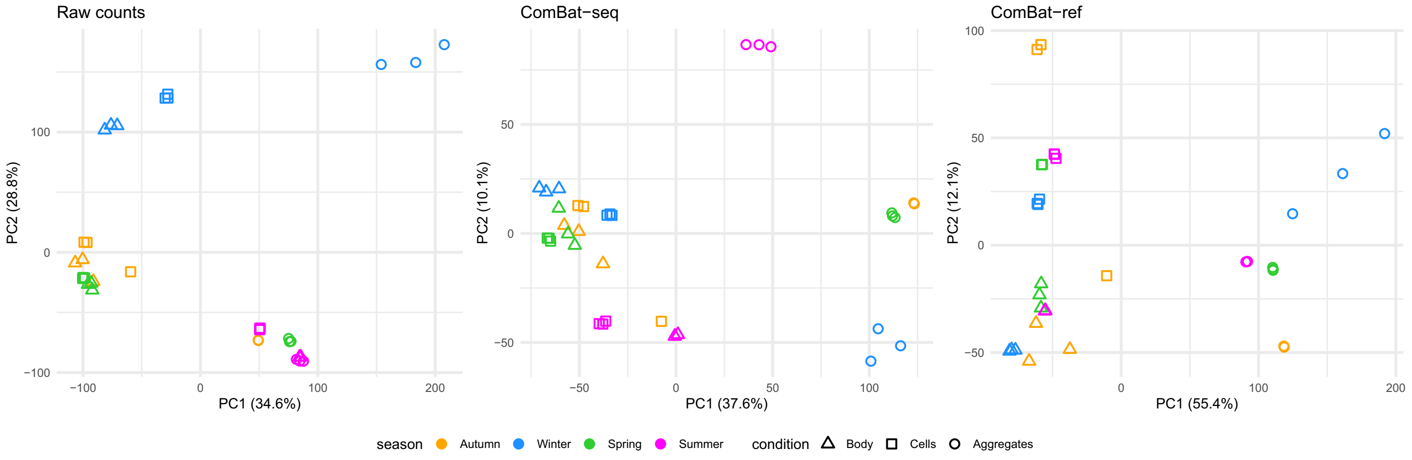

Figure S3. Differential expression volcano plots.

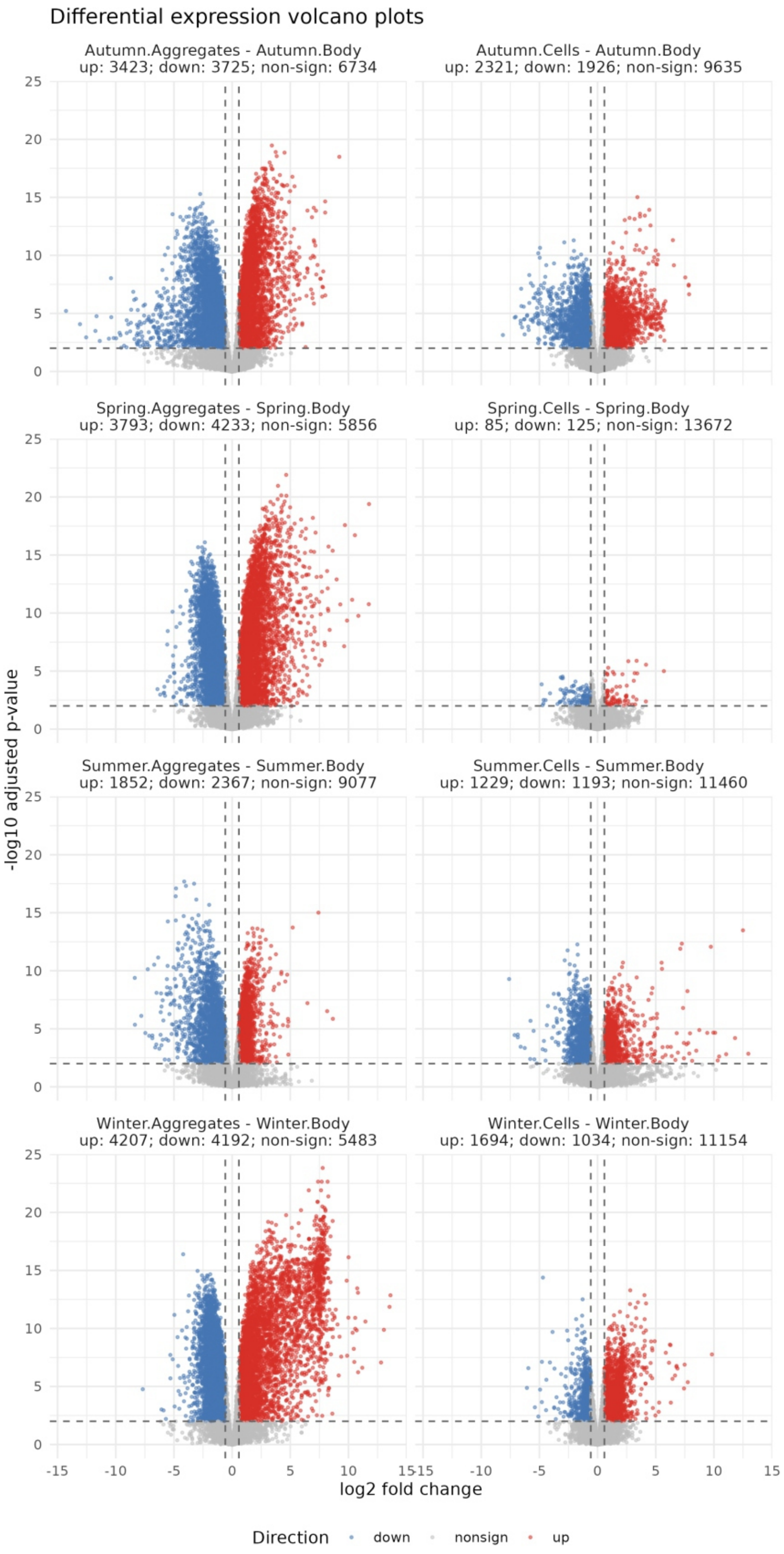

Figure S4. The examples of chromatograms for *Halisarca dujardini* body (A) and cells (B) samples. The peak of dopamine (DA) standard is marked with an arrow.
(C) Regression line for dopamine. Standard curves were linear from 1,56 to 50 pmol/ml dopamine. The coefficients of correlation (*r*) are higher than 0.999 indicating a good relationship between the peak area and the concentration in these concentration ranges.

A

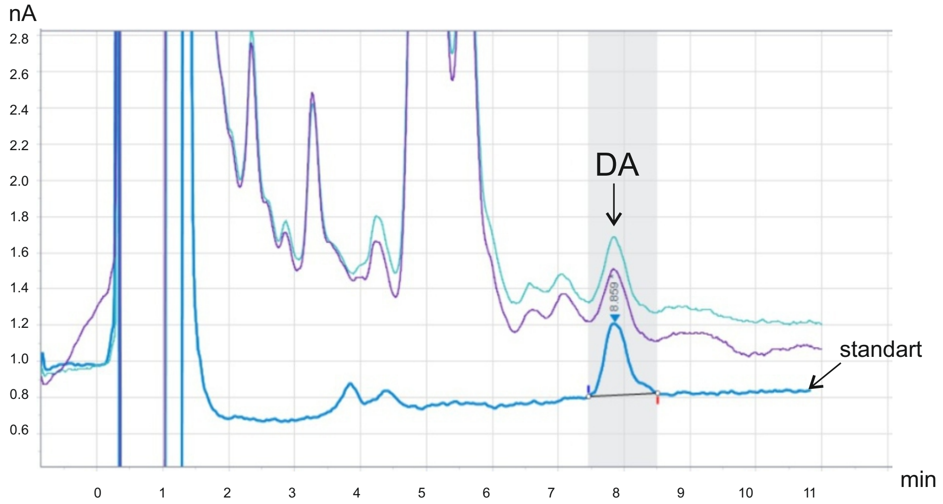

B

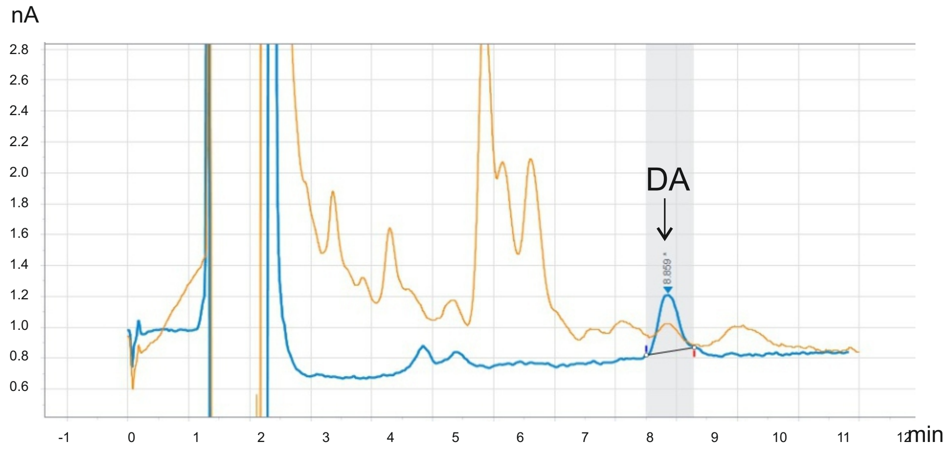

C

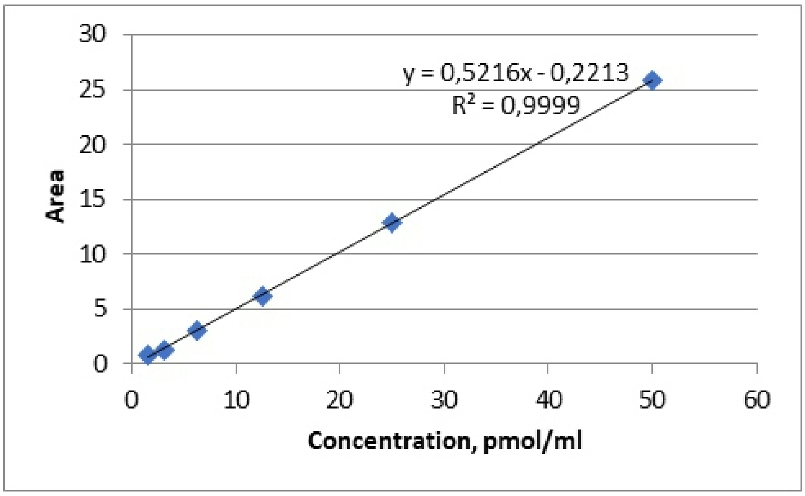

Figure S5. The examples of chromatograms for *Halisarca dujardini* (A, B) and seawater (C) samples. The peak of (DA) and noradrenaline (NA) standards are marked with an arrow.

A

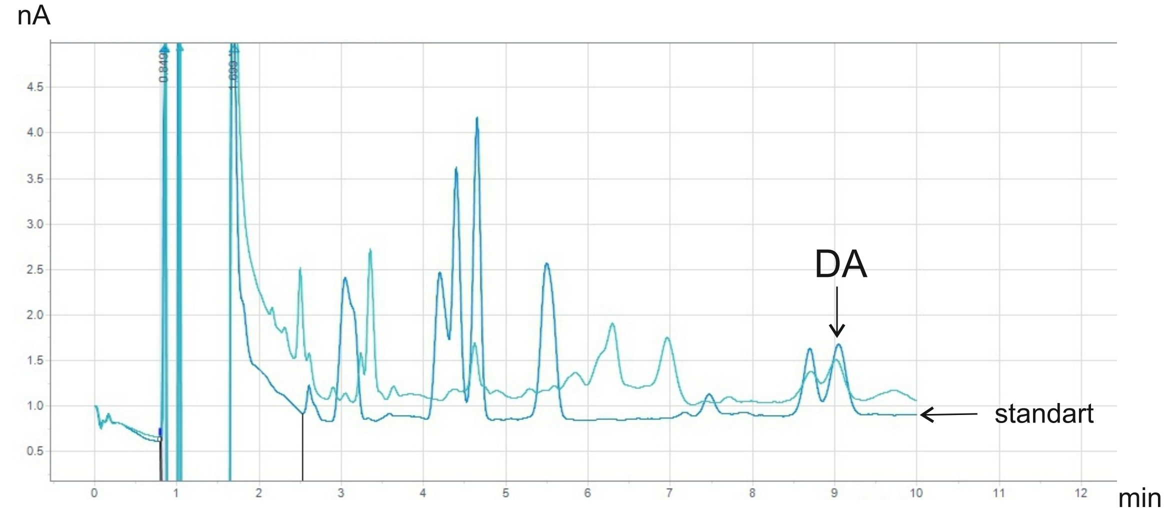

B

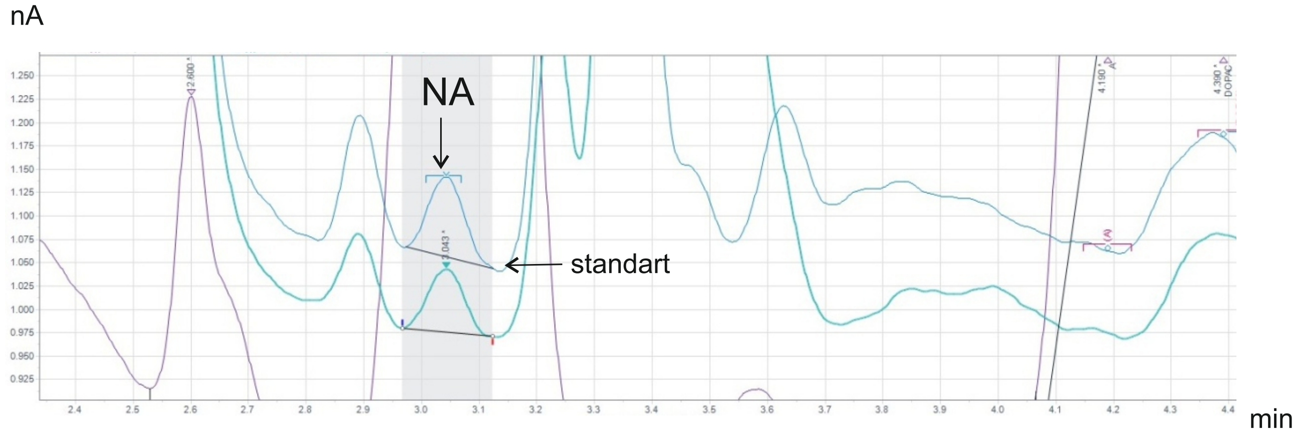

C

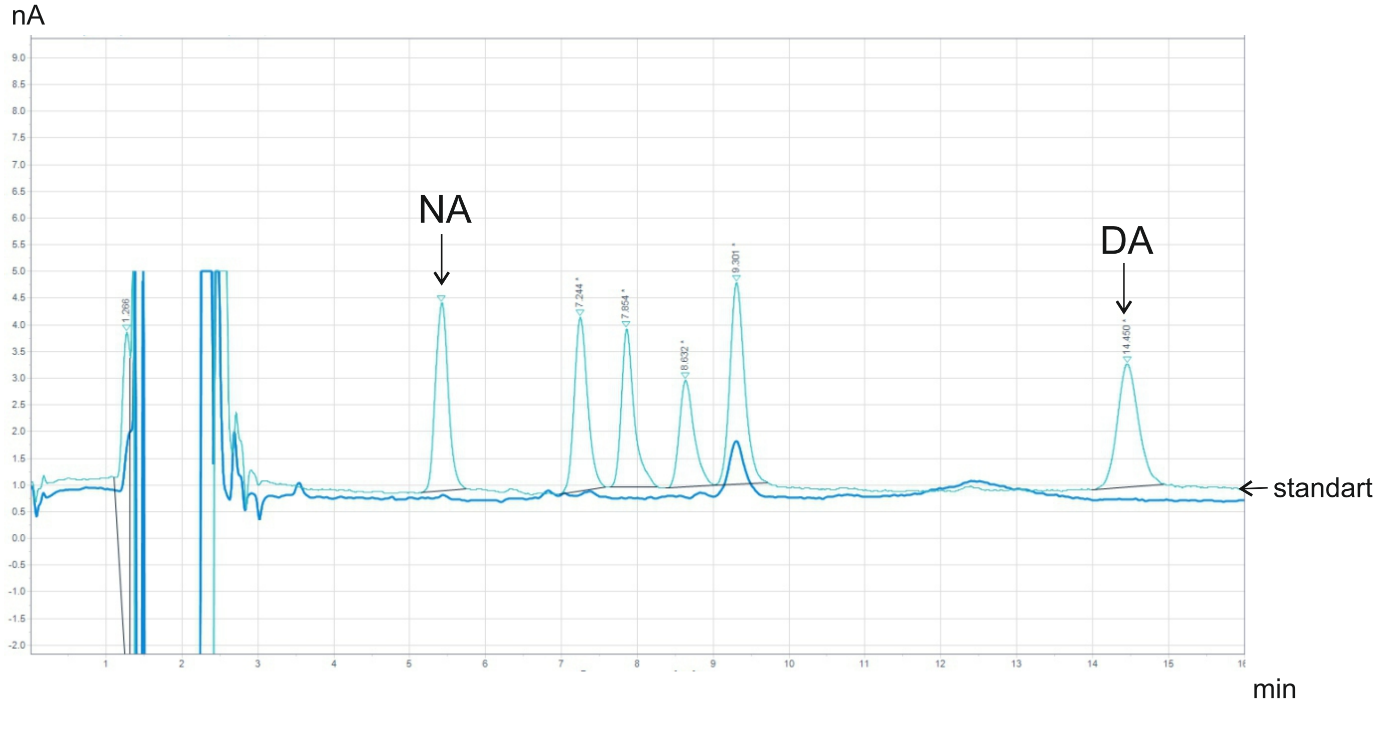

Figure S6. (A) The examples of chromatograms for *Sycon ciliatum* sample. The peak of dopamine (DA) standard is marked with an arrow.
(B) DA content (pM/mg) in *Sycon ciliatum* control sample and after incubation in 10 μM L-DOPA solution

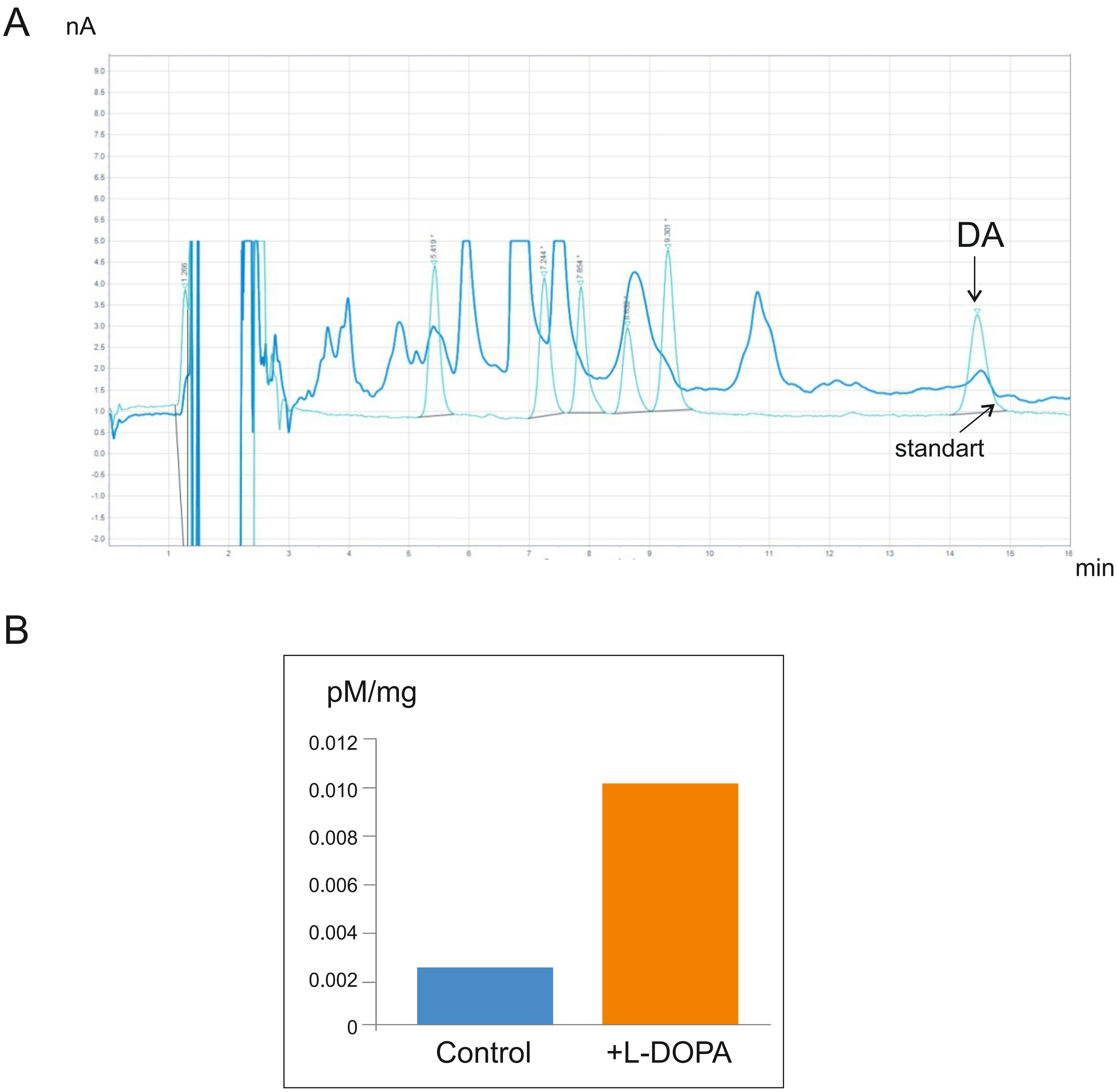

Figure S7. Phylogeny of metazoan aromatic amino acid hydroxylases. The tree was reconstructed using Maximum Likelihood inference of IQ-TREE with the best-fitting Q.yeast+I+R5 evolutionary model; node support was evaluated with ultrafast bootstrap; nodes with >95% support are marked with black dots, and the rest of support values are omitted; the lineages of tryptophan and tyrosine hydroxylases, which emerge in bilaterians from a broader group of phenylalanine hydroxylases, are highlighted.

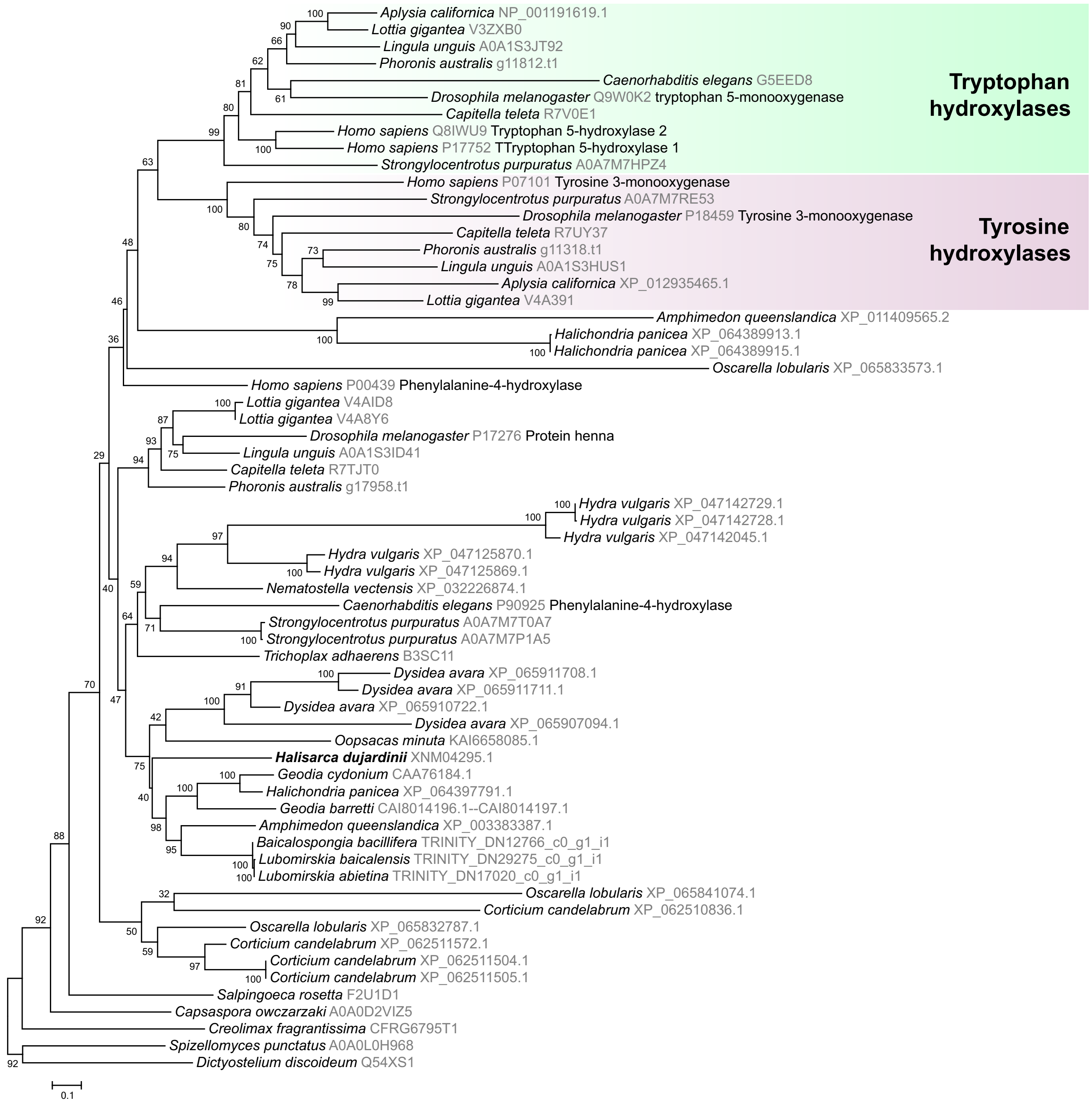

Figure S8. Phylogeny of sponge tyrosinases.
The tree was reconstructed using Maximum Likelihood inference of IQ-TREE with the best-fitting VT+R5 evolutionary model; node support was evaluated with ultrafast bootstrap.

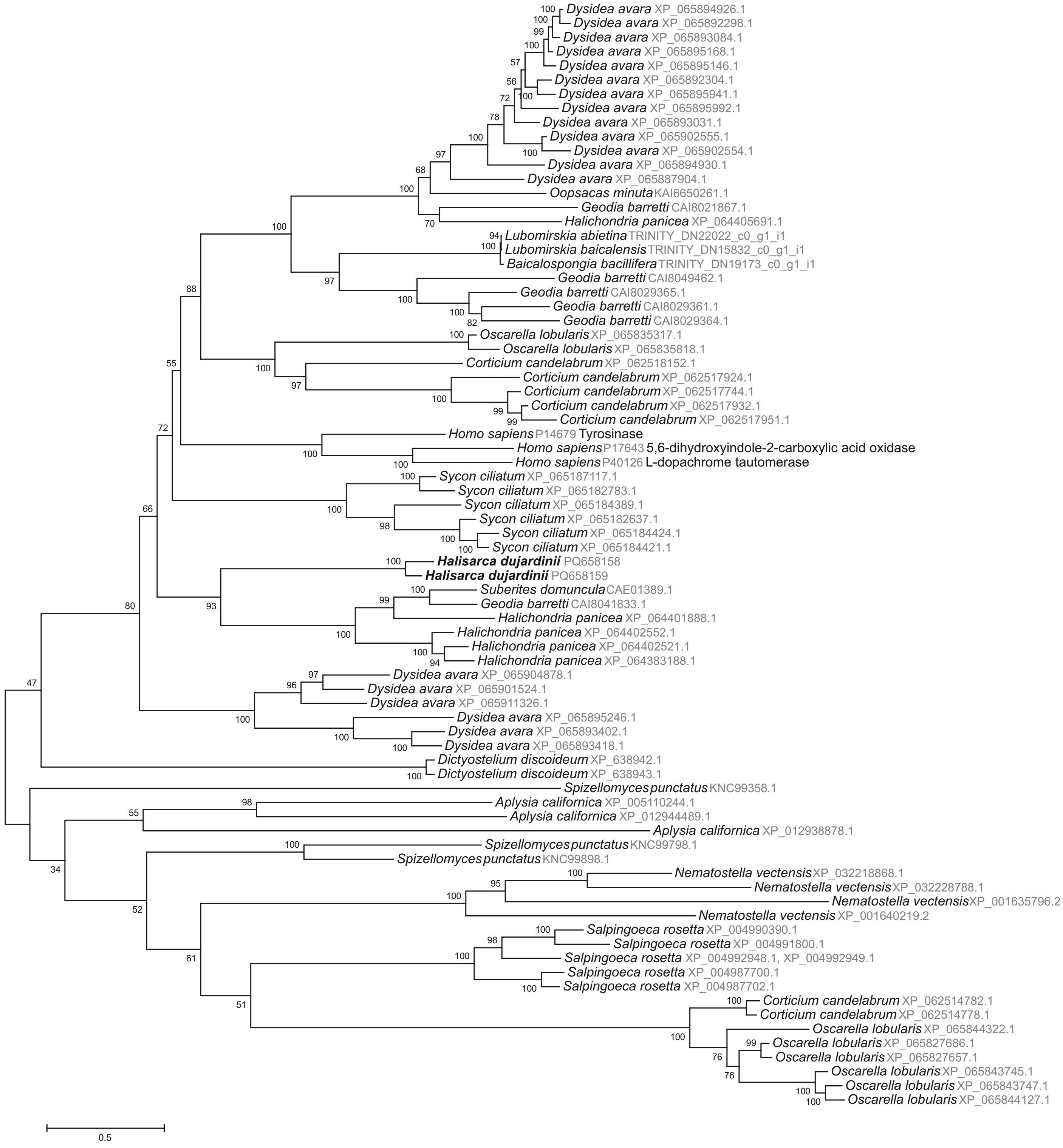

Figure S9. Domain structure of sponge and human tyrosinases.
The transmembrane domain (TMD), the signal peptide (SP), and the copper binding site (Cb(A) and Cu(B)) are shown.

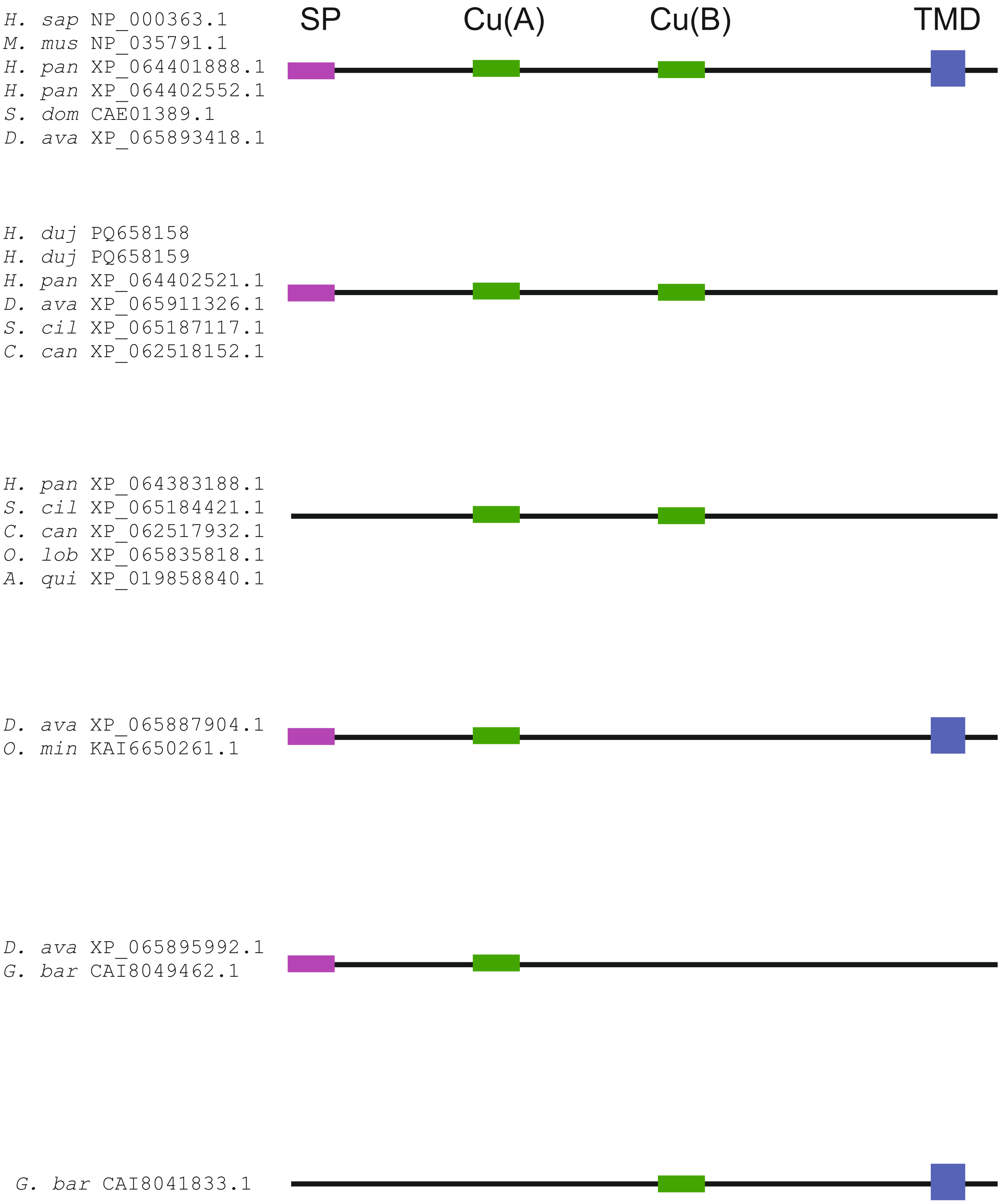

Figure S10. Phylogeny of group II pyridoxal-dependent decarboxylases.
The tree was reconstructed using Maximum Likelihood inference of IQ-TREE with the best-fitting Q.pfam+F+R6 evolutionary model; node support was evaluated with ultrafast bootstrap; nodes with >95% support are marked with black dots, and the rest of support values are omitted; bacterial sequences are labeled blue, and clades consisting entirely of prokaryotic representatives are collapsed (blue triangles).

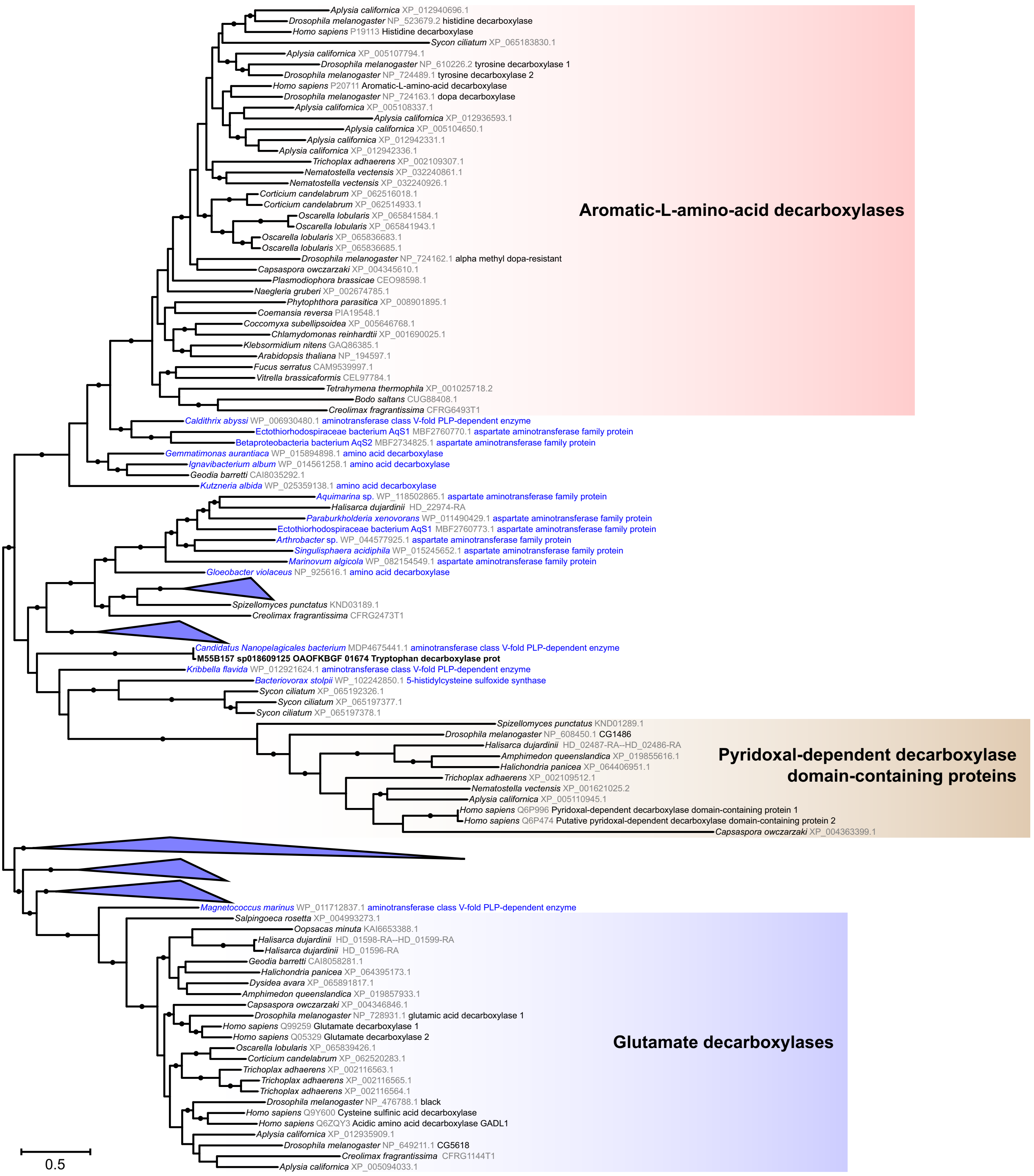

Figure S11. Phylogeny of DBH-like monooxygenases.

The tree was reconstructed using Maximum Likelihood inference of IQ-TREE with the best-fitting Q.pfam+R6 evolutionary model; node support was evaluated with ultrafast bootstrap; the common architecture of DBH-like monooxygenases is depicted in the upper right corner with the following domains: DoH – DOMON domain (PF03351), Cu2_monooxygen and Cu2_monoox_C – copper ion binding regions of copper type II, ascorbate-dependent monooxygenases (PF01082 and PF03712); the N-terminal signal peptide is marked red, and the C-terminal transmembrane region is depicted as a blue rectangle; the domain architectures of *Halisarca* DBH-likes are shown.

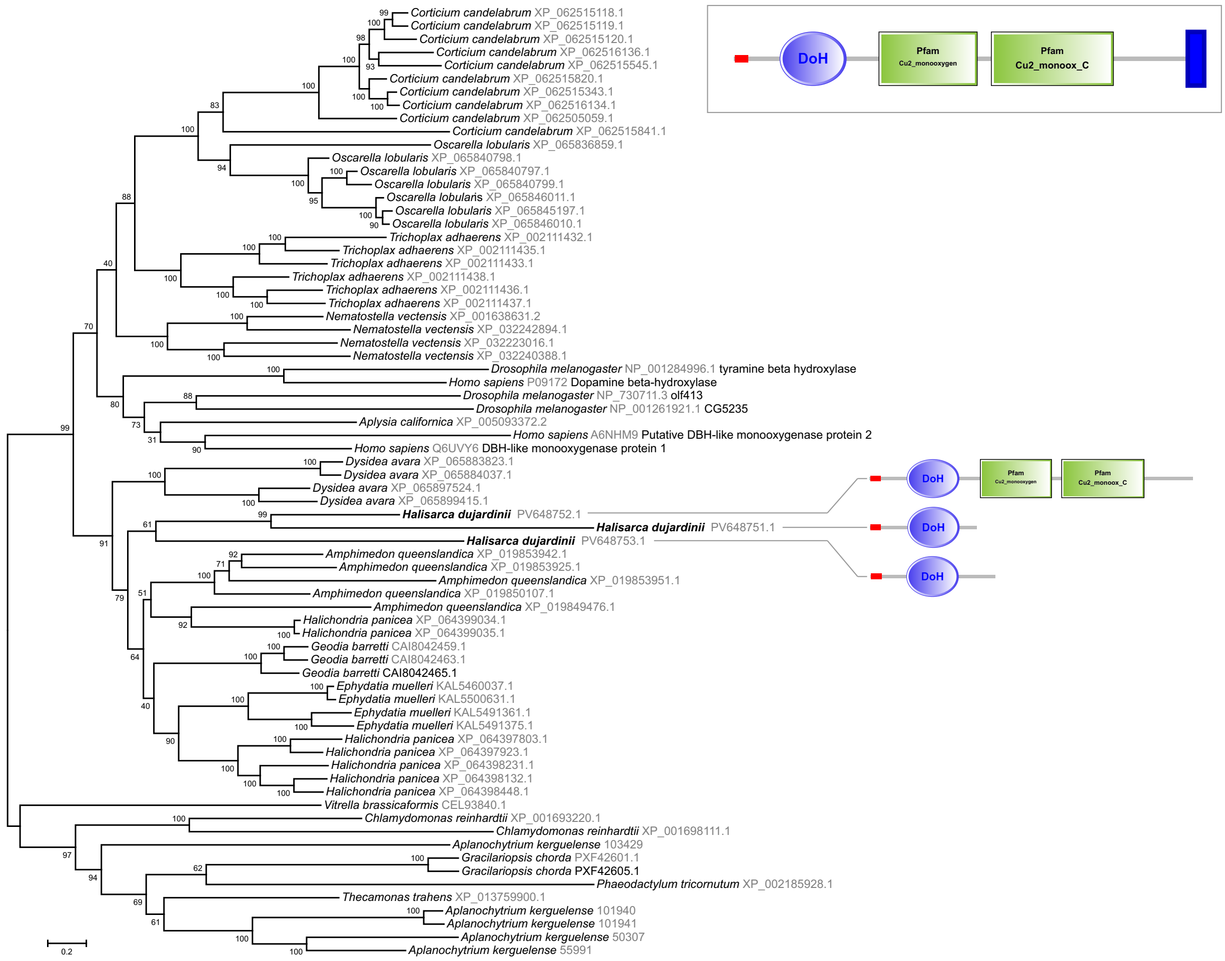

Figure S12. The alignment of DOPA_deC_like domaine (cd06450) for pdb|1JS6|A Chain A DOPA decarboxylase and bacterial decarboxylases. M55B157_sp018609125 - *H. dujardini*-associated bacteria AqS1 and AqS2 - bacterial symbionts of *A. queenslandica* (10.3389/fncir.2023.1250694). cd06450 domain was found using CCD (https://www.ncbi.nlm.nih.gov/Structure/cdd/). for M55B157_sp018609125 (E-value 1.15e-79), AqS1 (E-value 5.71e-58) and AqS2 (E-value 1.01e-56). The catalytic residue (lys) is shown by an asterisk, the pyridoxal 5'-phosphate binding site is shown by blue.

```
                                                                                                                                                                                                                         *
pdb|1JS6|A Chain A DOPA decarboxylase   GSASEATLVALLAARTKVVRRLQAASPGLTQGAVLEKLVAYASDQAHSSVERAGLIGGV---KLKAIPSDGKF-AMRASALQEALERDKAAGLIPFFVVATLGTTSCCSFDNLLEVGPICHEEDIWLHVDAAYAGSAFICPEFRHLLNGVEFADSFNFNPHKWLL
M55B157_sp018609125_OAOFKBGF_01674      SGGTVSNITALTAARERAFP--GSRQHGVVPGSATA----YCSADAHYSICRAIEVLGIGSQWLRPIPVDVNR-RMNPIALSHAIDDDIAQGRTPMVVVATSGTTLTGAIDPIDAIADVCDVHGVWLHIDGAYG--LPAAAVMPDAFTGLARADSVSVDAHKWMF
MBF2734825.1 B. bacterium AqS2          DSASSGTLAAVLTARERATG-WQGNEAGLAGGPPLR---MYYSRHAHASVPKAIMLAGLGRANAVAIDLDADG-AMDAGALERAIEADRAAGMKPAGVVATVGATSTGDADGLAATGAVVRRHGLYGHVDAAWAGSAALCPEHRGLLDGLEQWDSYLFNPHKWLG
MBF2760770.1 E. bacterium AqS1          DTASSATLAAVLTMRERALD-GKGNSRGLAGQPALR---IYASNEVHSSIDRALWFSGIGADNLVRIPTAGPMRGMILERLRDAIAADRAAGFLPAGIVAAVGGTSTGACDDIAAVSQVAQEESLYLHVDAAWAGSAMICPEFRSLWHGAEHADSIVLNAHKWLG
```

Figure S13. B6 metabolism in (A) HiC_scaffold_22 (o. Rhodospirillales_A; f. Casp-alpha2); (B) M55B157_sp018609125 (o. S36-B12; f. S36-B12; g. M55B157; s. M55B157_sp018609125); (C) UBA4486 (o. UBA4486; f. UBA4486).

A

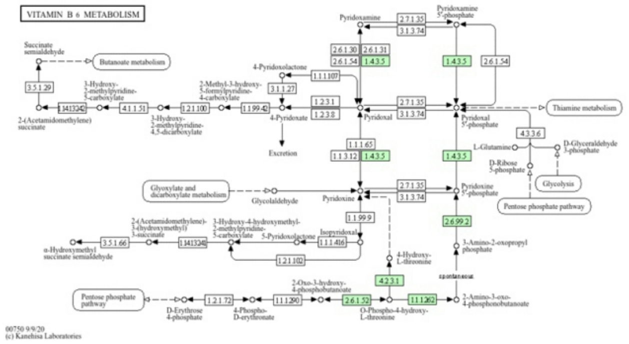

B

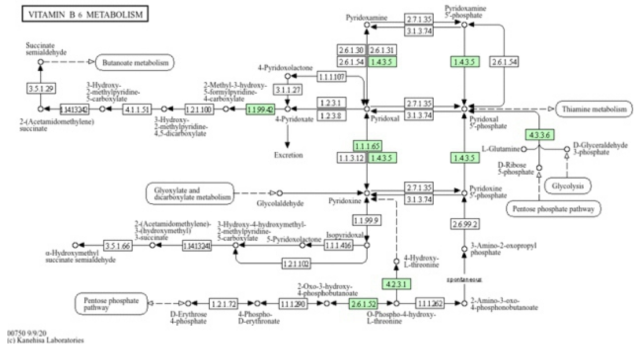

C

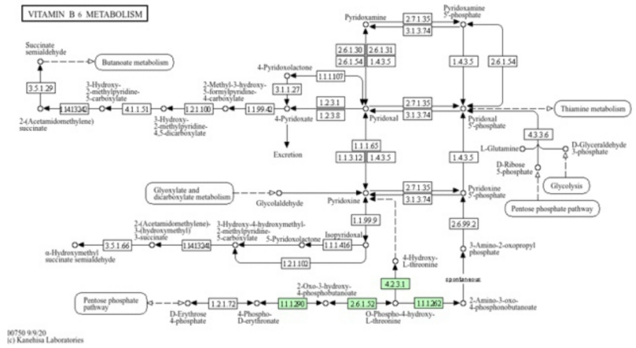

Figure S14. Phylogeny of animal rhodopsin-like G protein-coupled receptor family. The tree was reconstructed using Maximum Likelihood inference of IQ-TREE with the best-fitting mtInv+F+R10 evolutionary model; node support was evaluated with ultrafast bootstrap; nodes with >95% support are marked with black dots, and the rest of support values are omitted; the tree branches are colored according to the species the protein belongs to (bottom right), and the corresponding species affiliations are additionally encoded on the circular color strip; clades with high support that are comprised of sequences that belong to only one species are collapsed (triangles) when they contain more than ten members; available annotations for several groups of human receptors are provided on the rim of the diagram; the receptors of *Halisarca dujardini* are highlighted in red, and the four analyzed in the study are marked on the color strip 1-4 as they are referred to in text.

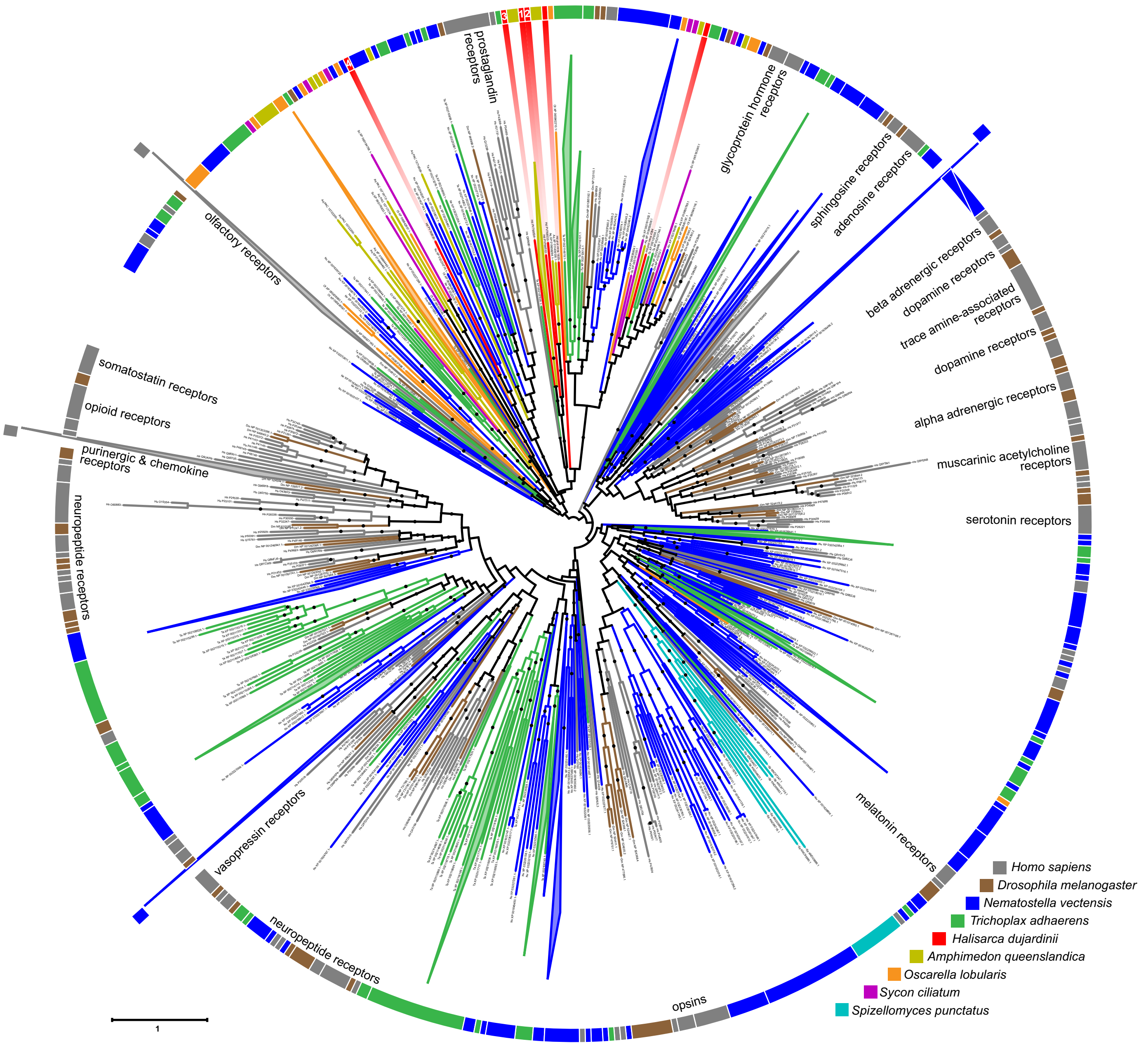

Figure S15. The 5-HT, NA and DA potential binding sites in galanin receptor type 3-like of *Amphimedon queenslandica*, predicted in MOE. Affinity to serotonin, NA, and DA are shown in the Table S4. The amino acids are numbered according to the sequence of each protein. The hydrogen bonds are shown as dotted lines.

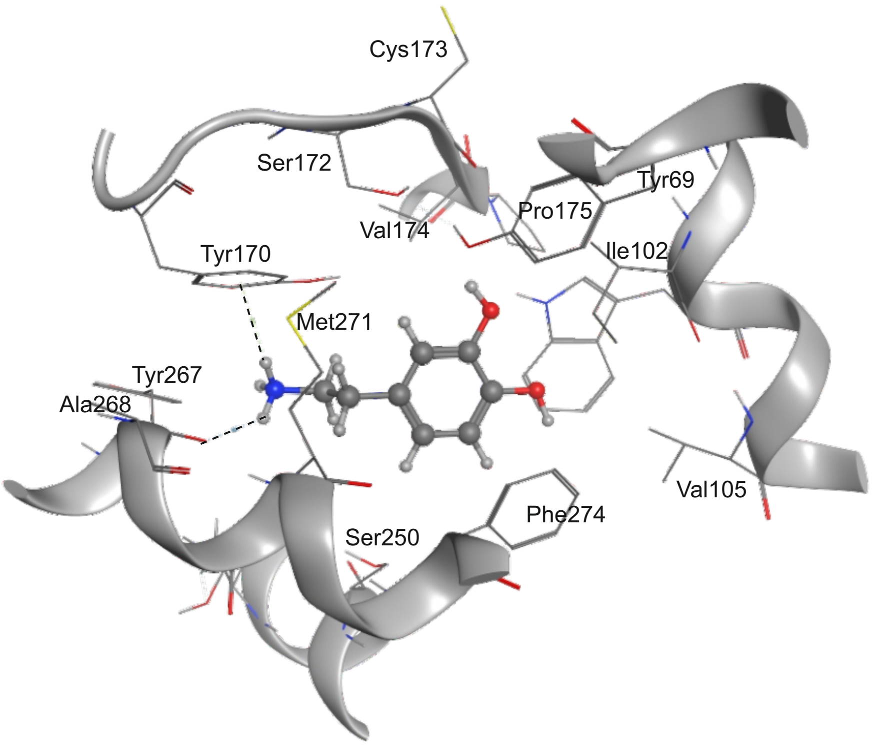

Figure S16.(A) UMAP visualization of of *Halisarca dujardini* G-protein coupled receptor 1-4 (GPCR 1-4) expression levels across single-cell clusters. (B) Dotplot illustrating the expression of G-protein coupled receptor 1-4 (GPCR 1-4) across single-cell clusters. Color represents the mean value of normalized expression of the gene in the cluster. Dot size represents fraction of cells that have non-zero level of expression of this gene.

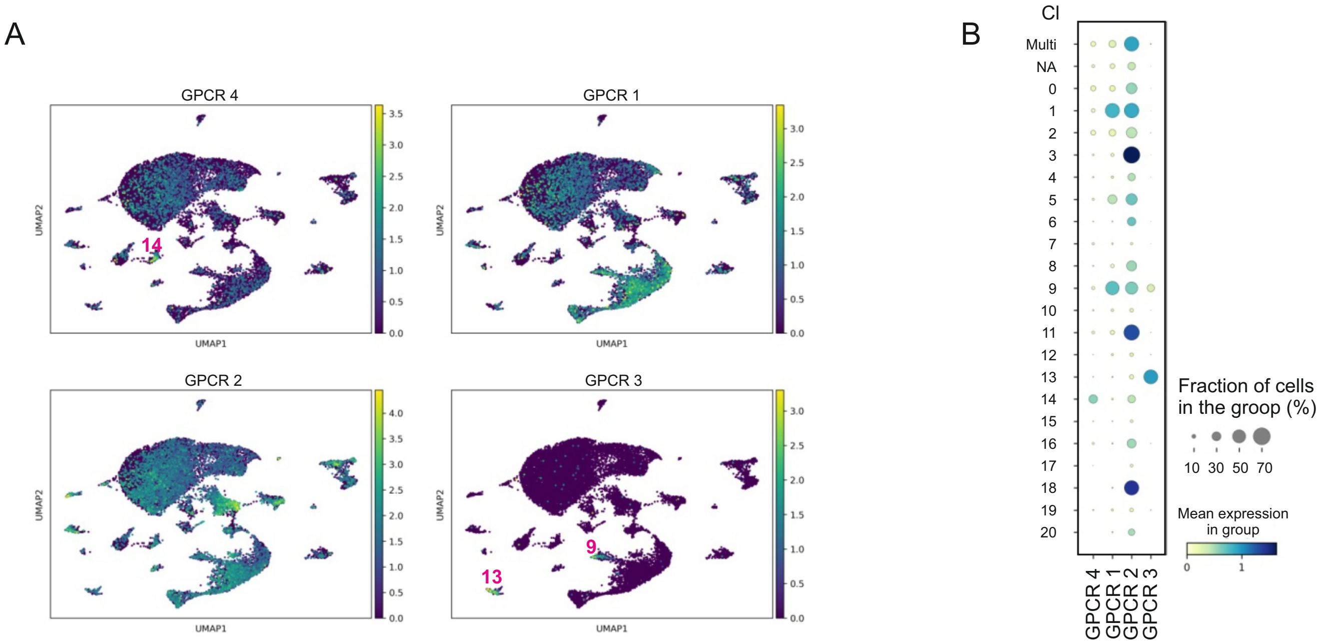

Figure S17. SDS-PAGE of *H. dujardini* protein lysates (60 µg of protein/well)
(A) Coomassie staining. The examples of the bands for mass spectrometric analysis.
(B) Fluorescence detection of MDC. 1 – control, 2 - MDC with 1 mM calcium chloride, 3 - MDC with 1 mM calcium chloride and 100 µM cystamine dihydrochloride, 4 - MDC. M - marker.
(C) Relative amounts (represented by the optical density) of MDC fluorescence normalized against the total amount of proteins. The data presentes as mean +- SEM in conventional units, with the optical density in the control taken as the unit. n=4, n reflects biological replicates.

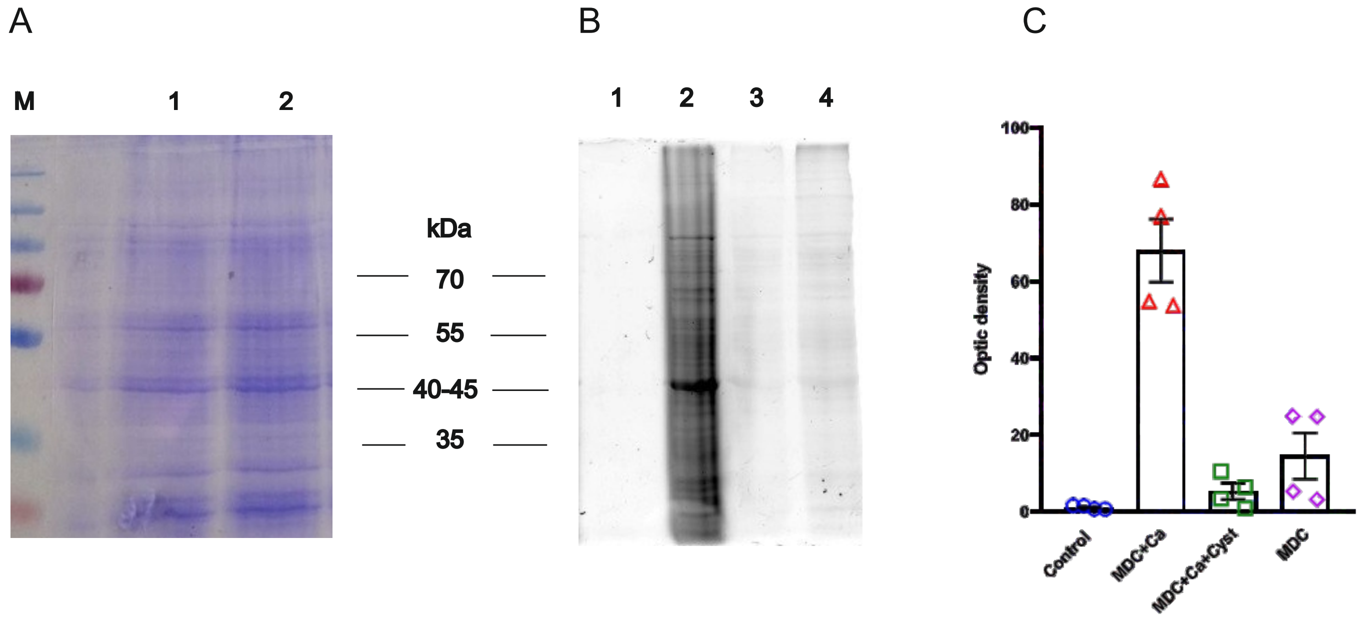

Figure S18. The mass-spectrometry fragmentation spectra for *H. dujardini* actin (QSX72278.1).

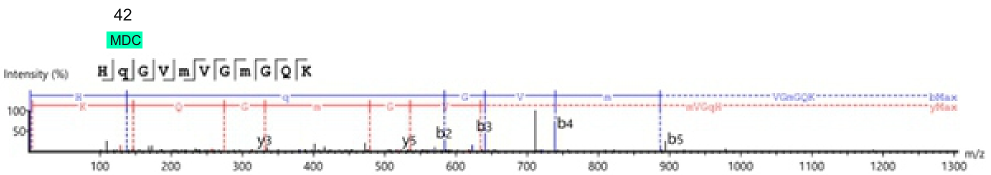

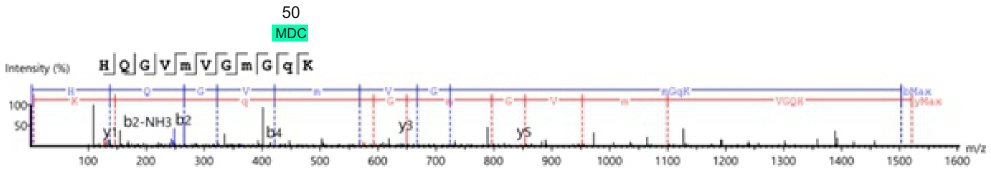

MDC MDC (+318.14)

Figure S19. The mass-spectrometry fragmentation spectra for (A) *H. dujardinii* twinfilin-1a and (B) actin (QSX72278.1) peptides shown in Table S13.

A

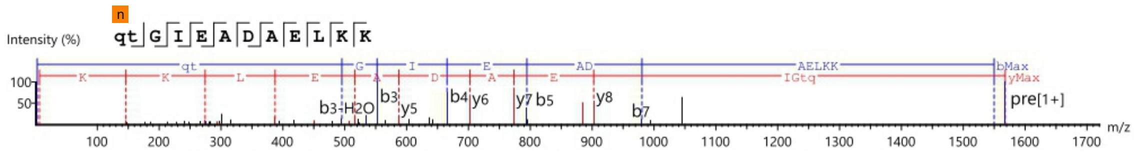

B

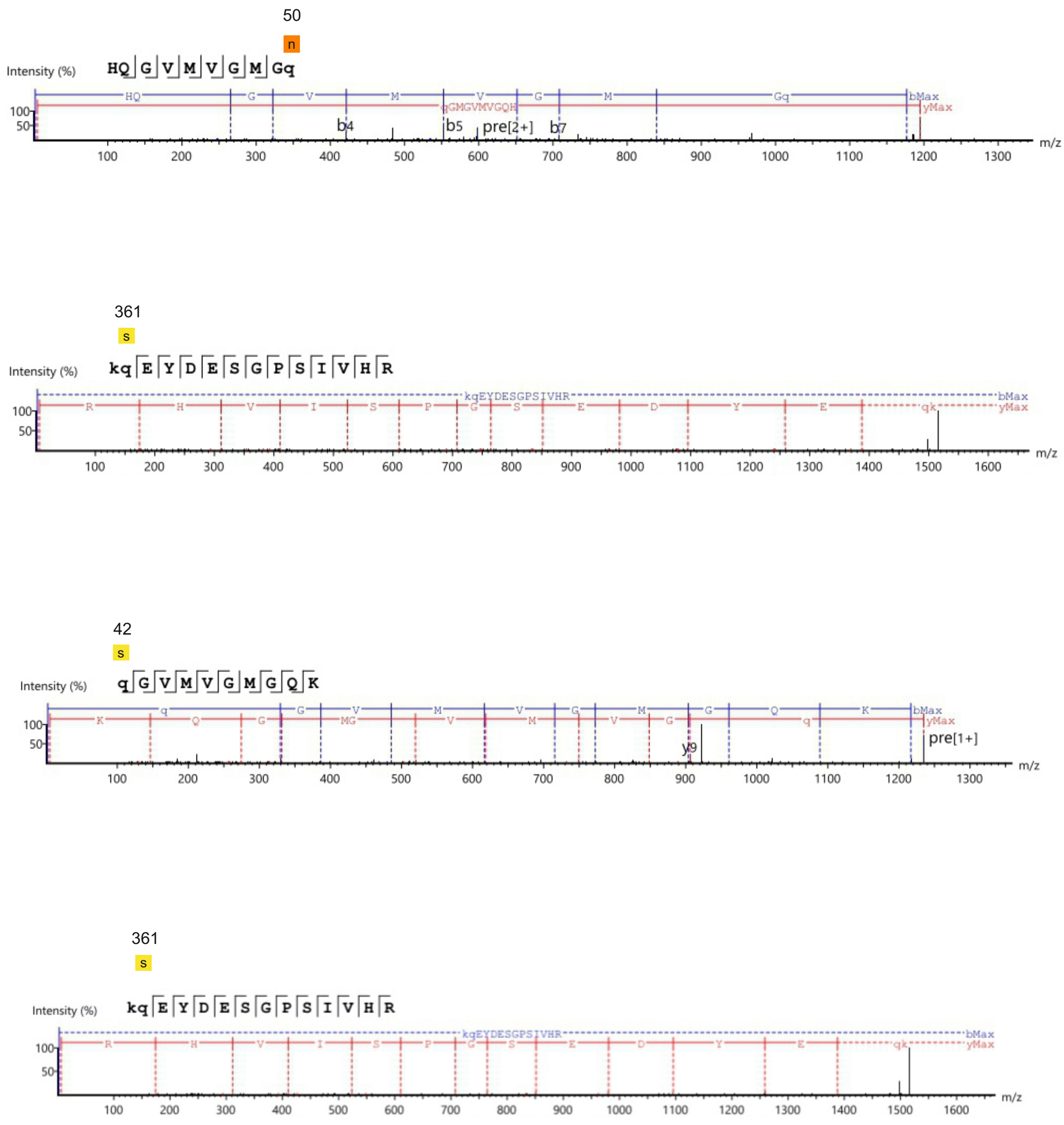

N Noradrenaline (+152.0)

S Serotonin (+159.07)

Figure S20. The mass-spectrometry fragmentation spectra for *Sycon ciliatum* gelsolin-like protein 1 (XP_065178474.1).

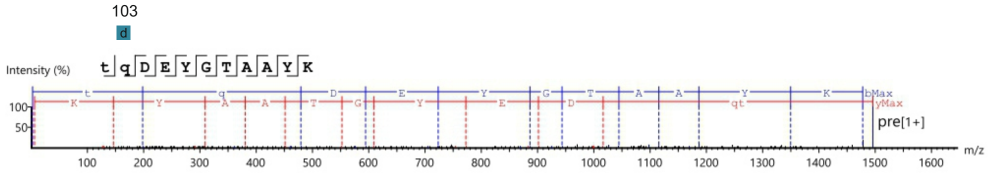

D Dopa (+136.05)
