# Supplementary material for: Evolutionary conservation of dopamine-mediated cellular plasticity in Arctic sponges (Porifera)
Source: Front Mol Biosci. 2025 Nov 17;12:1671771. doi: 10.3389/fmolb.2025.1671771 (PMC12665527; doi:10.3389/fmolb.2025.1671771)
Supplement: Supplementary file 9 [file Table11.docx]

Table S11. Extended mass-spectrometry data for *H. dujardini* and *S. ciliatum.*

| # | Organism | Description | Number of peptids | Number of proteins | Peptids with DOPA (Q) | Peptids with dopamine quinone (K) | Peptids with 3,4-dihydroxyphenylacetaldehyde (K) | Peptids with 5-HT  (Q) | Peptids with NA  (Q) |
| --- | --- | --- | --- | --- | --- | --- | --- | --- | --- |
| 1 | *H. dujardini* | Adult, January | 7383 | 1037 | 0 | 0 | 0 | 0 | 0 |
| 2 |  | Adult, February | 4674 | 860 | 0 | 0 | 0 | 0 | 1 |
| 3 |  | Adult, March | 2850 | 776 | 0 | 0 | 0 | 0 | 0 |
| 4 |  | Adult, March | 3284 | 941 | 0 | 0 | 0 | 0 | 0 |
| 5 |  | Adult, March | 3488 | 957 | 0 | 0 | 0 | 0 | 0 |
| 6 |  | Adult, March | 7424 | 1291 | 0 | 1 | 0 | 0 | 0 |
| 7 |  | Adult, May | 3037 | 534 | 1 | 1 | 1 | 0 | 0 |
| 8 |  | Adult, May | 3930 | 806 | 1 | 0 | 0 | 1 | 1 |
| 9 |  | Adult, August | 2150 | 367 | 1 | 0 | 0 | 0 | 0 |
| 10 |  | Adult, September | 5549 | 853 | 1 | 0 | 0 | 1 | 0 |
| 11 |  | Adult treated with L Dopa, September | 6040 | 859 | 1 | 0 | 0 | 0 | 0 |
| 12 |  | Adult, November | 5780 | 1041 | 1 | 0 | 0 | 1 | 0 |
| 13 |  | Adult, November | 2422 | 557 | 0 | 0 | 0 | 0 | 0 |
| 14 |  | Larva, June | 6746 | 1012 | 1 | 0 | 0 | 0 | 0 |
| 15 | S. ciliatum | Adult, March | 5853 | 1413 | 1 | 0 | 0 | 0 | 0 |
| 17 |  | Adult, March, treated with L-DOPA | 1161 | 1406 | 0 | 0 | 0 | 0 | 0 |
