# Supplementary material for: Evolutionary conservation of dopamine-mediated cellular plasticity in Arctic sponges (Porifera)
Source: Front Mol Biosci. 2025 Nov 17;12:1671771. doi: 10.3389/fmolb.2025.1671771 (PMC12665527; doi:10.3389/fmolb.2025.1671771)
Supplement: Supplementary file 10 [file Table6.docx]

Table S6. The closest homologs for *H. dujardini*  G-protein coupled receptors.

| *H. dujardini*  G-protein coupled receptor | The closest homologue | AlfaFold structure |
| --- | --- | --- |
| PV768532 | A0A1X7U348 | AF-A0A1X7U348-F1 |
| PV768533 | A0A1X7U4C3 | AF-A0A1X7U4C3-F1 |
| PV768534 | A0A1X7UKS4 | AF-A0A1X7UKS4-F1 |
| PV768535 | A0A7M5V649 | AF-A0A7M5V649-F1-v4 |
